# New flavors from old wheats: exploring the aroma profiles and sensory attributes of local Mediterranean wheat landraces

**DOI:** 10.3389/fnut.2023.1059078

**Published:** 2023-05-18

**Authors:** Sivan Frankin, Alon Cna'ani, David J. Bonfil, Vered Tzin, Kamal Nashef, Doron Degen, Yasmin Simhon, Marina Baizerman, Maria Itria Ibba, Héctor Ignacio González Santoyo, Cyntia Velazquez Luna, Jose Fausto Cervantes Lopez, Anomarel Ogen, B. Z. Goldberg, Shahal Abbo, Roi Ben-David

**Affiliations:** ^1^Institute of Plant Sciences, Agricultural Research Organization–Volcani Institute, Rishon LeZion, Israel; ^2^The Robert H. Smith Institute of Plant Sciences and Genetics in Agriculture, The Hebrew University of Jerusalem, Rehovot, Israel; ^3^Department of Food Sciences (UCPH-FOOD), Design and Consumer Behavior, University of Copenhagen, Frederiksberg, Denmark; ^4^Jacob Blaustein Center for Scientific Cooperation, Jacob Blaustein Institutes for Desert Research, Ben-Gurion University of the Negev, Midreshet Ben-Gurion, Israel; ^5^Gilat Research Center, Agricultural Research Organization, Gilat, Israel; ^6^French Associates Institute for Agriculture and Biotechnology of Drylands, Jacob Blaustein Institutes for Desert Research, Ben-Gurion University of the Negev, Midreshet Ben-Gurion, Israel; ^7^Global Wheat Program, International Maize and Wheat Improvement Center (CIMMYT), Heroica Veracruz, Mexico; ^8^Bread Holdings Inc.-GAIL's The Bread Factory, Bertinet, United Kingdom; ^9^The Mediterranean Food Lab, Tel Aviv, Israel

**Keywords:** wheat landraces, durum, wholemeal flour, aroma compounds, sensorial panel, sourdough bread, amino acids, organic acids

## Abstract

**Introduction:**

During the 20th century, the worldwide genetic diversity of wheat was sharply eroded by continual selection for high yields and industry demands for particular standardized qualities. A collection of Israeli and Palestinian landraces (IPLR) was established to represent genetic diversity, accumulated for ten millennia under diverse environments, which was mostly lost in this transition. As our long-term goal is to study this pre- Green Revolution genetic reservoir, herein we focus on its flour and bread quality and sensorial attributes.

**Methods:**

Initially, a database was built for the entire IPLR collection (n=901) holding both *Triticum durum* (durum wheat) and *T. aestivum* (bread wheat) which included genetic and phenotypic characterization of agronomic traits, grain and flour quality. Then, a representative subset of the IPLR was selected and compared to modern varieties for dough quality, rheology, aroma and taste using both whole and refined flours and breads. The sensory panel used 40 subjects who evaluated common protocol or sourdough breads made by four artisan bakers.

**Results:**

Results show modern durum cultivar C-9 had superior rheological properties (gluten index, elasticity, dough development time) as compared with landraces, while bread landrace 'Diar Alla' was markedly preferable for baking in relation to the modern cultivar Gadish. Baking tests and subsequent sensory evaluation clearly demonstrated a preference toward refined breads, apart from whole breads prepared using sourdough starters. In bread wheat, loaves baked using landrace flour were scored higher in several quality parameters, whereas in durum lines, the opposite trend was evident. Loaves baked from landraces 'Diar Alla' and to a lesser extent 'Hittia Soada' presented a markedly different aroma from the control loaves prepared from modern flours, both in terms of overall compositions and individual compounds, including classes such as pyranones, pyrazines, furans and pyrroles (maltol). Modern lines, on the other hand, were consistently richer in terpenes and phenylpropanoids. Further analysis demonstrated a significant association between specific aroma classes and sensory attributes scored by panelists.

**Discussion:**

The findings of the study may help advance new niches in the local wheat market aimed at health and nutrition including adapting durum varieties to the bread market and developing flavor-enhanced wholemeal breads.

## 1. Introduction

Wheat is the most cultivated crop in temperate regions (1) with global preliminary forecast production of 784 MT for 2023 (https://www.fao.org/worldfoodsituation/csdb/en/) and is widely used for human food as well as livestock feed. The most widely cultivated wheat species is bread wheat (*Triticum aestivum* L), accounting for over 95% of wheat currently grown worldwide. Durum wheat (*Triticum durum* Desf.) accounts for 5% of wheat cultivated area and traditionally was mostly grown in the Mediterranean Basin (2). Wheat landraces have been a main source for food and energy since the cradle of agriculture and only few decades ago these heritage cultivars were replaced with semi-dwarf elite varieties. Specifically in Israel, this transition also completed the process of bread wheat becoming the major dryland crop at the expense of durum and barley. In the Mediterranean Basin, in the past and still today, durum wheat is a staple crop and the basis for traditional foods such as couscous, bulgur, and flat pita bread. Durum has also been used to make leavened bread such as Pane di Altamura (historically documented to 37 B.C.) (3) or in combination with barley to produce semi-leavened bread typical to the Maghreb (Morocco and Tunisia predominantly) (4). Landraces grown and maintained locally at the community or family level also preserve traditional recipes and unwritten “know-how” regarding the best use of these varieties in the traditional cuisine. Such “know-how” might include understanding suitability for specific dishes, flavor, texture, shelf life, etc.

Flavor, which encompasses both taste and aroma properties, is one of the most important attributes of foods in terms of consumer preferences and perception (5). Food aroma is a complex mixture of low molecular weight volatile organic compounds (VOCs) at different compositions. These mixtures are partitioned between the food matrix and the headspace, depending on the ambient conditions and volatile identity (6). Volatiles can be detected by smelling (orthonasal) or ingesting (retronasal) (7). Taste, the other constituent of flavor, is defined by molecules that impart sweet, bitter, sour, salty, and umami sensations and which interact with oral receptors of the gustatory system (8). In bread, aroma is a product of the complex interaction between raw materials, the biological leavening agent, and the baking process: it comprises metabolites readily present in flour and precursors that are modified by endogenous enzymes (autolysis), degraded/modified during fermentation and formed during baking (9). Bread taste is mainly defined by the presence of non-volatile sweet imparting compounds (sugars and some amino acids), sour compounds (organic acids), and to a lesser degree, bitter, umami, and salty conferring metabolites (10). The type of compounds readily available in flours can, to some extent, be retained in breads and contribute to their aroma. Since milling involves selective removal and enrichment of several parts of the kernel, it also changes the aroma of the flour considerably, resulting in both pleasing- and off-flavors in whole flours (10–15). Aroma compounds generated during baking are formed predominantly in crusts and depend directly on non-volatile precursors present in the dough, i.e., sugars and amino acids. The Maillard and caramelization reactions use precursors liberated during fermentation and are accelerated under elevated temperatures (9). Maillard is a set of complex redox reactions that involves interaction between carbonyl groups from reducing sugars and compounds containing an amine group (mainly amino acids) (9–11, 13, 14, 16–18).

Wheat landraces have been highlighted numerous times as an important source for genetic diversity, leading to isolation and integration of genes conferring favorable agronomic phenotypes in various traits including abiotic and biotic stress resistance. A new genetic reservoir of landraces from Israel and Palestine (IPLR collection) was recently constructed (*n* = 901) (19) and a representative subset of the IPLR collection was tested for its agronomic performance under multi-environments (20). Landraces are also a potential source for improving grain quality and nutritional traits in wheat, including phenolics in general, and antioxidants (carotenoids and tocols in particular) (21). However, only a handful of studies have looked into ancient lines and landraces as a source for unique flavor profiles (1, 22–25). For instance, Ficco et al. (26) tested the aroma of flours and breads of a durum landrace and modern lines, and determined that the genetic background was critical, albeit secondary to milling degree and leavening agents. Ruisi et al. (17) compared breads made from durum landraces to those made of modern lines using VOC analysis and sensory characterization. The authors demonstrated high variability in aroma among bread loaves made from landrace grains and reported similar sensory scores of these breads compared with bread baked with flour of modern varieties, with some even exceeding those of the modern references. Starr et al. (25) performed a comprehensive aroma analysis of a wide panel of landrace and modern lines, and concluded that landrace flours were richer in esters, alcohols, and furans. Overall, landraces have the potential of encompassing new flavors that could be utilized to improve the flavors of breads either directly or through breeding with modern lines. Wheat quality improvement plays an important role in all breeding programs (27). Specific quality requirements include mainly protein quantity, gluten strength and extensibility, pigment color, kernel size, and dough performance (28). The principal goal of this study was to characterize the quality attributes and the aroma profile of refined and wholemeal flour and to preform sensorial evaluation of bread made of *T. durum* and *T. aestivum* landraces from a subset of the IPLR collection in comparison to modern cultivars.

## 2. Materials and methods

### 2.1. Plant material

The Israeli Palestinian landraces collection, 901 lines in total [*T. aestivum* (*n* = 173) and *T. durum* (*n* = 728)], consists of landraces and modern Israeli cultivars as a control group (n = 55). The complete IPLR collection was grown in a common garden setup in 2018–2019 at Volcani center, Rishon LeZion, Israel. For a full description, see Frankin et al. (19). From each accession, 20 g seeds were sampled and used for further grain quality analysis. A representative landraces panel constructed of landraces of *T. durum* (*n* = 7), landraces of *T. aestivum* (n = 6), modern cultivars of, *T. durum* (*n* = 2, cv. C-9, and cv. Solet), and modern cultivars of *T. aestivum* (*n* = 2, cv. Ruta and cv. Gadish) was chosen from the IPLR collection (20) for further examination of bread sensorial profiles and rheology. This panel, 17 in total was grown during two consecutives cropping seasons (2018–2019 and 2019–2020) at Gilat Experimental Station, located in Negev, south of Israel, under a semiarid environment with supplementary irrigation (262 mm and 363 mm in total during 2018–2019 and 2019–2020, respectively). Plots were sown on November 12, 2018, and November 17, 2019, and were harvested in June 2019 and 2020, respectively [for full description, see Frankin et al. (20)]. Nitrogen fertilization was applied at pre-planting and herbicides and fungicide were applied as needed to keep plots free from weeds and pests. Out of the representative panel, six landraces and two modern cultivars were chosen for post-harvest evaluation and rheology analysis at Gilat bread laboratory (Table 1).

**Table 1:** Grain and rheological characteristic of refined flour over 2 years (2019–2020).

**Name**	**Group**	**Specie**	**Season**	**GY**	**TKW**	**HW**	**PRO**	**GLU**	**GI**	**EXT**	**P**	**L**	**W**	**DDT**	**WAC**	**S**
				t ha^−1^	g	kg hl^−1^	%	%		%	mm	mm	10^−4^ J	min	%	min
8238	Landrace	*T. durum*	'19	2.4	46.6	80.6	14.5	18.5	10.9	65.5	111	42	156	2.12	65.4	2.37
			'20	2.8	46.4	75.1	17.0	25.5	5.9	58.3	111	40	146	2.5	65.4	2.83
Hittia Soada	Landrace	*T. durum*	'19	2.9	47.8	78.6	15.6	22.5	4.4	61.4	128	53	200	2.18	68.4	1.07
			'20	2.9	43.3	78.5	16.5	23.5	6.4	56.6	110	56	177	2.53	67.7	2.3
Gaza	Landrace	*T. durum*	'19	2.8	38.0	74.7	13.6	17.5	5.7	68.4	86	43	123	2.68	61.5	4.02
			'20	1.9	38.1	72.2	14.7	23.0	23.9	64.0	91	51	141	2.22	63	2.9
C-9	Modern cultivar	*T. durum*	'19	-	-	-	-	-	-	-	-	-	-	-	-	-
			'20	5.1	43.2	81.5	13.0	29.0	82.8	59.9	189	45	348	5	63.5	22.5
Lubnani Kirsa	Landrace	*T. aestivum*	'19	3.2	45.0	77.3	12.9	28.5	71.9	55.1	55	119	162	3.88	56.4	10.1
			'20	3.2	42.2	77.9	14.6	31.5	17.4	56.0	65	77	163	4.52	55.1	11.5
Diar Alla	Landrace	*T. aestivum*	'19	3.0	34.5	78.8	13.6	29.0	57.0	64.2	104	73	238	17	63	5.13
			'20	2.8	35.0	76.9	14.4	31.0	40.3	61.1	111	79	304	21.9	61.8	18
Palestinskaya	Landrace	*T. aestivum*	'19	2.4	27.9	74.3	15.5	25.5	25.5	60.7	72	32	95	2.93	56.6	29.1
			'20	1.7	30.0	76.1	14.8	27.0	16.8	50.0	48	40	65	2.4	54.3	30.4
Gadish	Modern cultivar	*T. aestivum*	'19	4.3	49.4	83.6	14.4	33.0	19.7	74.5	76	64	191	5.88	57.9	4.07
			'20	6.0	45.5	82.4	13.6	30.5	47.6	69.3	58	112	219	6.98	55	2.12

GY, grain yield; TKW, thousand kernel weight; HW, test weight; PRO, protein content; GLU, wet gluten; GI, gluten index; EXT, white flour extraction; P, strength; L, elasticity; W, alveograph index; DDT, dough development time; WAC, water absorption content; S, stability.

### 2.2. Grain and flour evaluation

To characterize the quality attributes of the Israeli Palestinian landraces, we conducted a preliminary quality-screening test for all the IPLR collection (*n* = 901) at CIMMYT grain quality lab, El-Batan, Mexico. The same protocol was applied for the complete IPLR collection. Evaluation included grain test weight (HW), thousand kernel weight (TKW), both measured with the digital imaging system SeedCount SC5000 (Next Instruments, Australia) using the software and digital image application included in the equipment. Grain protein (12.5% moisture basis) content (PRO) was measured using near-infrared spectroscopy (DA 7200 NIR, Perten Instruments, Sweden), with a calibration validated using Leco^®^/Dumas method (correction factor: 5.83. Equipment FP828 Leco Instruments, St Joseph, Michigan, USA). Flour yellowness was obtained as the b^*^ value of a Minolta color meter model CR-410 (Konica Minolta, Japan). Gluten strength was estimated through the sodium dodecyl sulfate sedimentation test (SDS) using 1 g flour and following the protocol described by Peña et al. (29).

For the representative panel (*n* = 8), grain quality evaluation was performed at the Bread Quality Laboratory at Gilat Research Center for the two consecutive seasons. NIR spectrometry (Foss NIR System Model 6500), which measures reflectance in the 400–2,498 nm wavelength region, was used to determine PRO, after in-house calibration against protein content (N% × 5.7) determined by the micro-Kjeldhal method and the AACC method 46-13 (30). Grain samples were ground in a Lab Mill 3,100 using a standard 0.8-mm sieve (Perten Instruments, Sweden). The resultant wholemeal flour was used immediately or placed in an air-tight container, from which samples with the same dry weight, equal to 10 g of the meal, were taken to wash out the gluten. The washing-out was conducted with a Glutomatic 2,200 (Perten Instruments) according to AACC method 38-12 (AACC, 2,000) enabling wet gluten (GLU) and gluten index (GI) determination. Before milling grains samples (3 kg) were tempered to 16% moisture at 22°C for 24 h. Milling was performed with a Quadrumat Sr. (Brabender Instruments, Germany) enabling refined flour extraction (EXT) determination. Refined-flour dough quality was evaluated using two methods, a Chopin alveograph (France) in accordance with AACC method 5430-A1194 (AACC, 2000) to determine dough strength and elasticity [Alveograph index (W), dough tenacity (P) and extensibility (L)], and a Brabender^®^/ICC/BIPEA farinograph (Brabender Instruments, Germany) that measures dough development time (DDT), consistency (C), water absorption (WAC), and stability (S).

### 2.3. Aroma compounds profiling

Whole flours were milled using a Mockmill 200 (Wolfgang Mock GMBH, Germany); refined flours were milled using an AQC806S laboratory mill (Agromatic, Switzerland), sifted with a 250 μm mesh sieve. Samples (*n* = 3, 1 gr) were placed in 20 ml SPME glass vials (Chrom4, Germany) containing 1 gr of NaCl and 7 ml of a 20% (w/v) NaCl solution. *Iso*butylbenzene (10 mg L^−1^, Sigma-Aldrich, Israel) was supplemented as internal standard. Prior to analysis, vials were incubated for 15 min at 60°C within the built-in oven of a PAL COMBI-xt autosampler (CTC Analytics AG Switzerland) to release free volatiles into the headspace. A 10 mm long SPME fiber, assembly 50/30 μm, divinylbenzene/carboxen/polydimethylsiloxane (Supelco, USA) was inserted into vial's headspace for 30 min at 60°C for volatile extraction. The fiber was then desorbed for 10 min at 250°C within the inlet of a 7890A GC (Agilent, USA) equipped with an VF-5MS 10 m EZ-guard capillary column (30 m × 0.25 mm inner diameter, 0.25 μm film thickness), coupled to a 5977B MS detector (both Agilent, USA). Helium was the carrier gas in a constant rate of 1 mL min^−1^. Analysis (splitless mode) was performed under the following conditions: 1 min of isothermal heating at 40°C, followed by 6°C min^−1^ oven temperature ramp to 250°C. Ionization energy was 70 eV with a mass acquisition range of 40–400 *m/z* and a scanning rate of 6.34 spectra s^−1^. Retention index (RI) was calculated by running C8–C20 *n*-alkanes (Sigma-Aldrich, Israel) under the same conditions listed above. Compounds were identified using Wiley 10 with NIST 2014 mass spectral library data using the Mass Hunter software package (version B.08.00, Agilent, USA). Further identification of major compounds was based on a comparison of mass spectra and the retention index. Where possible, compounds were identified using authentic standards (Sigma-Aldrich, Israel), analyzed under the same conditions. Quantitative evaluation was performed using the internal standard (ISTD, *iso*butylbenzne), which was analyzed in increasing concentrations to generate a calibration curve. Detected peak areas in bread and flours samples were normalized to that of the ISTD and the value (expressed in μg) was extracted using the known concentration of the ISTD. The odor activity values (OAVs) for individual compounds were calculated as the ratio of compound's concentration in either flour or bread to the corresponding odor threshold in water (based on the literature and online sources cited in Supplementary Tables S2, S3).

### 2.4. Primary metabolites analysis

For evaluating basic taste metabolites, 100 mg of ground flour (whole or refined, same as described for aroma profiling, *n* = 3–4) was mixed with 1 ml of pre-cooled mixture of 2.5:1:1 MeOH:chloroform:Milli-Q water (v/v/v) supplemented with ribitol and ^13^C_6_ D-sorbitol (Sigma-Aldrich, Israel) as internal standards. The rest of the procedure was done following a previously described protocol (31) with minor modifications. In brief, the top 300 μL of hydrophilic layer was collected and dried in a vacuum. For derivatization, 40 μL of 20 mg/mL methoxyamine hydrochloride (Sigma-Aldrich, Israel) was added, dissolved in pyridine, and incubated for 2h in an orbital shaker at 37°C. Next, N-methyl-N-(trimethylsilyl) tri-fluoroacetamide (MSTFA), including an alkane standard mix in a volume of 77 μL, was added to each sample, followed by a 30 min incubation in an orbital shaker at 37°C. Finally, 1 μL of the sample was injected into the Agilent 5977B GC-MS [instrument specifications and running method are as described previously (31)]. Compound identification was done as listed above, and peak areas were normalized to that of ribitol and sorbitol.

### 2.5. Bread making

Bread making was conducted separately for each of the lines in two forms: refined and wholemeal flours. In 2020 (flour from 2018–2019 season), all loaves were prepared by one baker following the AACC protocol (method 10–10.03) (30) as follows. Ingredients: 250 g flour, 3.1 g salt, 2.5 g yeast, 1.4 g sugar, and 156.3 g water. The dough was slow kneaded over 2 min for coupling, then followed by 8 min kneading. The dough was placed in a proofing chamber for 1 h before kneading and shaping. Then, a second proof of 1 h before baking. Weight and volume were measured 1 h after baking (data not shown). In the following year (2021, flour from 2019–2020 season), the flour was divided between four artisan bakers. One baker followed the same conventional protocol and three artisanal bakers baked “free hand” sourdough loaves, each of them based on their independent personal protocols. Each iteration (2020 and 2021) was conducted once, with the 2^nd^ year comprising of one session dedicated to bread wheat and second to durum wheat.

### 2.6. Sensory evaluation

Sensory evaluation of breads made from modern or landrace flours was conducted in two consecutive years (2020–2021). In 2020, the session was held at the Gilat Research Center and the breads were evaluated by 17 panelists; the results of this session are presented in Supplementary Figure S1. In 2021, loaves of bread or durum wheats were evaluated separately for two consecutive weeks. In each session, the sensory evaluation was conducted over 3 h focusing on 20 loaves of either refined or whole flours. Both events were held at the Stybel Ltd training center (“Ad-Halom” mills, Ashdod, Israel). Panelists (*n* = 40) were screened and chosen based on their expertise and included bakers, food technologists, millers, wheat breeders, and academia representatives, all of which are highly familiar with bread quality. Tasting was blind and samples were letter coded. The evaluation of loaves in all three panels (of both years) was based on individual questionnaires with the following parameters (in each, a scale of 1–10 was given, representing attribute intensity or likeliness): crust color; pore size; general odor; sweet odor; rancid odor; nutty odor; sweet taste; sour taste; fruity flavor; bitter taste; typical flavor; good mouthfeel; doughy; juicy; airy; crust flavor; complexity and flavor richness; taste of more; and general score. The strictly hedonic-related questions were separated from the sensory attributes and omitted from further statistical analyses. The data collected from the tasters enabled comparison between the different lines, flours (refined/wholemeal), and sourdough/yeast starters, similar to the analysis done with the Gilat sensory panel during the first season (Supplementary Figure S1).

### 2.7. Statistical analyses

Descriptive statistics was applied on the entire IPLR collection to illustrate distribution of grain quality parameters. Principal components analysis (PCA) was performed based on the quality distribution and auto-correlated variables were removed from the analysis. Student's t and Dunnet's multiple comparison tests were executed using “R” package “DescTools” (29). All other statistical analyses (including two-way ANOVA and PCA) were performed using JMP^®^ ver.pro 16.0 statistical package (SAS Institute, Cary, NC, USA).

## 3. Results

### 3.1. Grain quality of the IPLR collection

**Figure 1:**
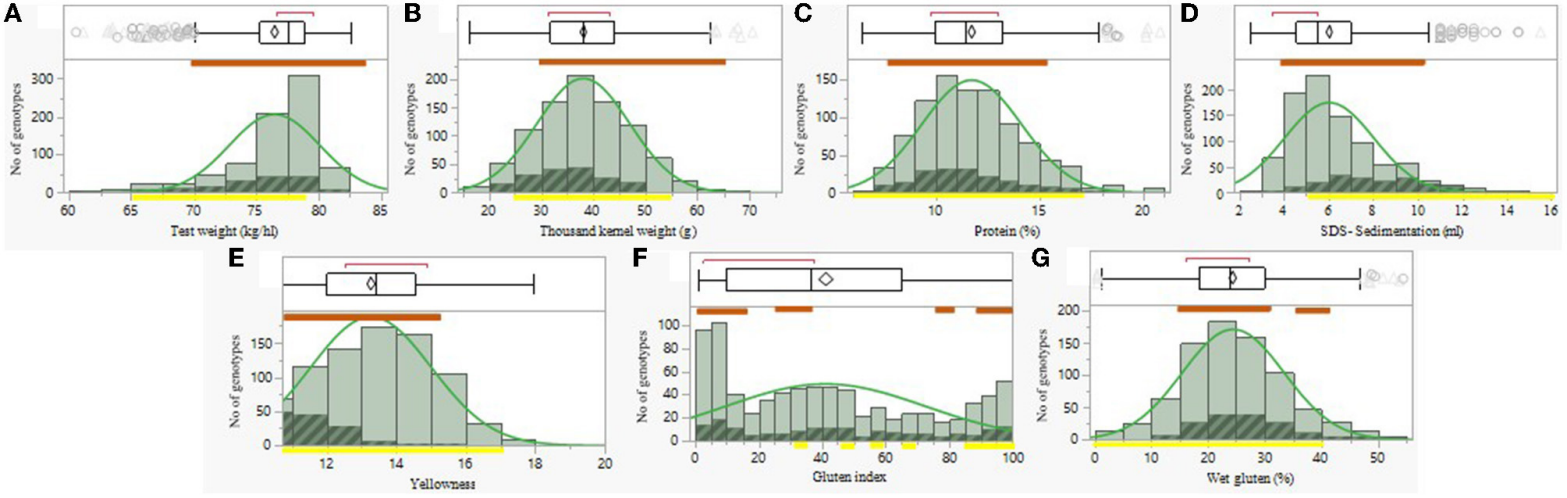
Distribution of grain quality compounds of the IPLR collection. Durum landraces (gray), bread wheat landraces (diagonal dashed line), subset landraces (brown horizontal line), and modern cultivars (yellow horizontal line). Test weight **(A)**; thousand kernel weight **(B)**; grain protein content **(C)**; sodium dodecyl sulfate sedimentation volume **(D)**; yellowness **(E)**; gluten index **(F)**; wet gluten **(G)**.

The IPLR collection represents a wide variation in quality parameters as described in Figure 1 and Supplementary Figure S2. The distribution of grain quality components values of the IPLR collection [*T. durum n* = 728, (gray color) and *T. aestivum n* = 173, (diagonal dashed line)] underline the diversity among landraces in comparison to modern cultivars [*n* = 55, (yellow horizontal line)] in all parameters: HW, TKW, PRO, SDS, yellowness, GI, and GLU (Figure 1). The subset panel of the landraces (brown horizontal line) represents this variation in small scale and expresses the potential of this germplasm for quality-oriented breeding programs. Higher test weight and yellowness values in the IPLR collection compared to modern control might have resulted from the high portion of *T. durum* accessions characterized by larger grain size. Within the IPLR collection, bread wheat landraces had higher SDS-sedimentation and relatively higher GLU compared with durum landraces. In modern cultivars, the gluten index score ranged between 65–100 (with three exceptions), which is within the Israeli industry standard range for bread making (32). The majority of the landraces, however, had GI <40 which classify them as animal feed grade grains. This is also expressed in higher SDS-sedimentation values in modern cultivars. PCA of grain quality parameters explained 62.2% of the variation in these traits and further supports these trends with partial discrimination of elite modern cultivars from the wide IPLR collection (Supplementary Figure S2). This is evident along PC2 (25.1%) which is positively loaded with GI and SDS, and negatively loaded with Yellowness (Supplementary Figure S2). Ploidy level discrimination is also evident diagonally, based on the negative association between SDS (high in hexaploids) and TKW (high in tetraploids) (Figure 1 and Supplementary Figure S2).

### 3.2. Rheology characteristics

Rheology characteristics of refined flour were evaluated from 2 years field trials (2018–2019, 2019–2020) for protein content (PRO), wet gluten (GLU), gluten index (GI), white flour extraction (EXT), strength (P), elasticity (L), alveograph index (W), dough development time (DDT), water absorption content (WAC), and stability (S) and are represented in Table 1 together with mean grain yield (GY), thousand kernel weight (TKW), and test weight (HW) values. As might be expected, the GY of modern cultivars C-9 and Gadish were 2-fold compared to the landraces total mean (5.1 and 5.15 t/ha, respectively, compared to total mean of 2.67 t/ha). Among the *T. durum* lines, the landrace “8238” had the highest thousand kernel weight in both years. “Hittia Soada” was prominent in L and WAC. Out of the three durum landraces, “8238”, and “Hittia Soada” had higher TKW, PRO, and WAC mean values in comparison with modern C-9. The latter cultivar excelled in most parameters (GY, HW, GLU, GI, P, W, DDT, and S); however, it should be taken into account that this particular cultivar was analyzed only in 2020. Within bread wheat, cv. Gadish excelled in high GY, TKW, HW, GLU, EXT, and DDT in both seasons. The landraces “Diar Alla” and “Lubnani Kisra” had a higher gluten index (57.0 and 71.9, respectively) than the modern reference Gadish (19.7) in the first season. This tendency reversed in the following season with Gadish (GI = 47.6), “Lubnani Kisra” (GI = 17.4), and “Diar Alla” (GI = 40.3). “Lubnani Kisra” had the highest elasticity score in 2019 (L = 119 mm) followed by Gadish (L = 112 mm in 2020). “Diar Alla” was prominent in strength (*P* = 104 mm and 111 mm in two consecutive seasons), alveograph index (W = 238 and 304 10^−4^J), dough development time (DDT = 17 and 21.9 min), and water absorption content (WAC = 63% and 61.8%). “Palestinskaya” had the highest stability (S = 29.1 and 30.4 min) (Table 1).

### 3.3. The aroma and taste profiles of flours milled from wheat landrace accessions

**Figure 2:**
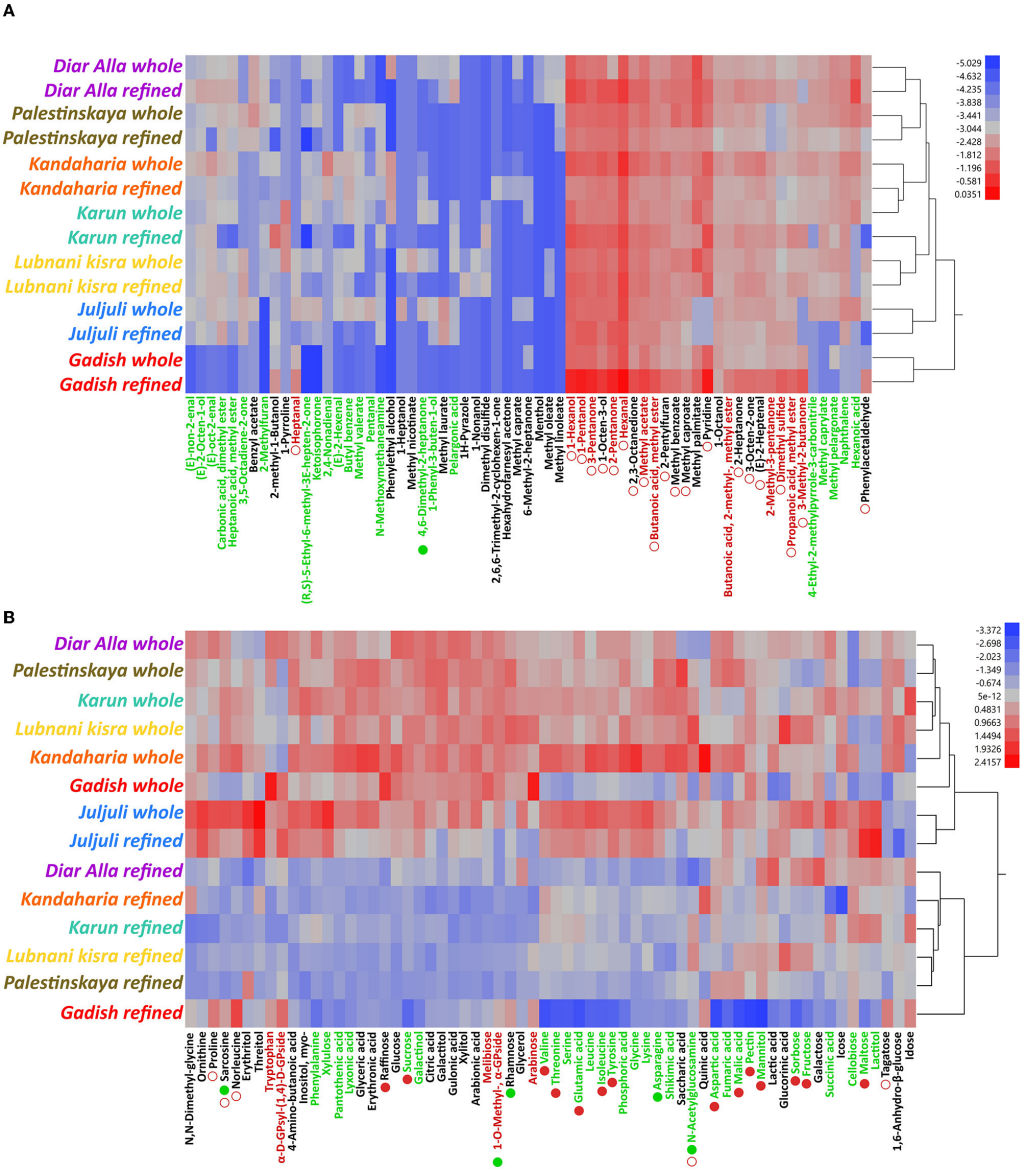
Aroma and taste compounds in *Triticum aestivum* flours (2019–2020). Figure present 2-way hierarchical clustering models (Ward's method, data is log_10_ transformed), of aroma **(A)** and taste-related compounds **(B)**. Metabolite concentrations are represented by an increasing color gradient from blue to red. Compounds in green font are significantly overrepresented in landraces; those marked by red font are overrepresented in the modern reference line. Full or empty bullets under the name denotes compounds that are either higher in whole or refined flours, respectively; bullet color represents whether this was evident in a landrace or a modern line, as mentioned previously. Dendogram and hierarchical clustering analysis were generated and visualized using JMP (version 13.2.0).

In order to evaluate whether landraces could potentially contain unique flavors, we milled whole and refined flours from nine wheat landrace lines (six *T. aestivum* and three *T. durum*) as well as two modern cultivars that served as reference for each crop. These flours were then analyzed using headspace solid phase micro extraction coupled with gas chromatography mass spectrometry (HS-SPME-GCMS) to elucidate the aroma makeup and a targeted trimethyl silylation followed by GCMS to measure basic taste compounds (sugars, amino acids, and organic acids). A total of 87 aroma compounds were identified across all flours. Of these, most were classified as lipid oxidation and fatty acid derivatives (sub-grouped into alcohols, esters, acids, aldehydes, and ketones; ~81% in average), followed by nitrogen containing compounds (pyridines, pyrazoles, amines, and pyrrolines, ~9.5%), phenylpropanoids (~4%), terpenoids (~3%), furans (~1.5%), and sulfur containing compounds (~0.5%, Supplementary Figures S3a–d). Aliphatic compounds (straight-chain/cyclic alkanes, alkenes, and alkynes) were omitted due to their negligible contribution to flavor, resulting in 68 compounds in total. Using a 2-way ANOVA model, we considered two factors: milling type and genotype (genetic background, i.e., whether a line is classified as modern or a landrace), as well as their interaction, when assessing the novel flavor potential of landraces. The flour aroma of *T. aestivum* lines was predominantly affected by the genetic background (i.e., landrace or modern) followed by milling and the interaction between both model factors (Figure 2A and Supplementary Table S1). Landraces were more closely related to each other than to the modern Gadish in terms of their overall aromas, underlined by higher proportions of furans, terpenes, and phenylpropanoids, as well as fatty acid-related methyl esters (carbonic acid, dimethyl ester; heptanoic acid, methyl ester; methyl caprylate; methyl pelargonate and methyl valerate, Figure 2A and Supplementary Table S1). However, the majority of overrepresented volatile compounds in either landrace or modern lines had no distinct classifications or sensory descriptors (a more detailed comparison between individual landraces and reference Gadish can be found in Supplementary Table S2). Interestingly, the milling × genotype effect was found to be significant almost exclusively in the modern cultivar. As such, compounds that were higher in Gadish compared to landraces were also more abundant in refined flours compared to wholemeal flours. Landrace grains, on the other hand, accumulated a set of compounds regardless of milling type (Figure 2A and Supplementary Figure S1). Taste constituents of the flours included a wide range of sugars (hexoses, pentoses, phospho-sugars, sugar alcohols, and sugar acids, as well as some di- and tri-saccharides) amino acids and their derivatives, and organic acids. As opposed to aroma profiles, the concentration of basic taste compounds in *T. aestivum* flours were mainly shaped by the milling process: whole flours had higher concentrations of almost all tested metabolites apart from a handful of compounds (Figure 2B and Supplementary Table S1). The genetic background of the line (i.e., landrace or modern) or the interaction between genotype and milling had relatively lower impacts. Landraces were markedly richer in free amino acids (except for tryptophan), organic acids, and several sugars important for fermentation including sucrose, fructose, and maltose. Levels of many taste-related metabolites were higher in whole compared to refined flours, but only in the modern Gadish and not in landraces, indicating that the interaction between milling and genotype was more pronounced in Gadish, similar to the aroma results (Figure 2B and Supplementary Table S1).

**Figure 3:**
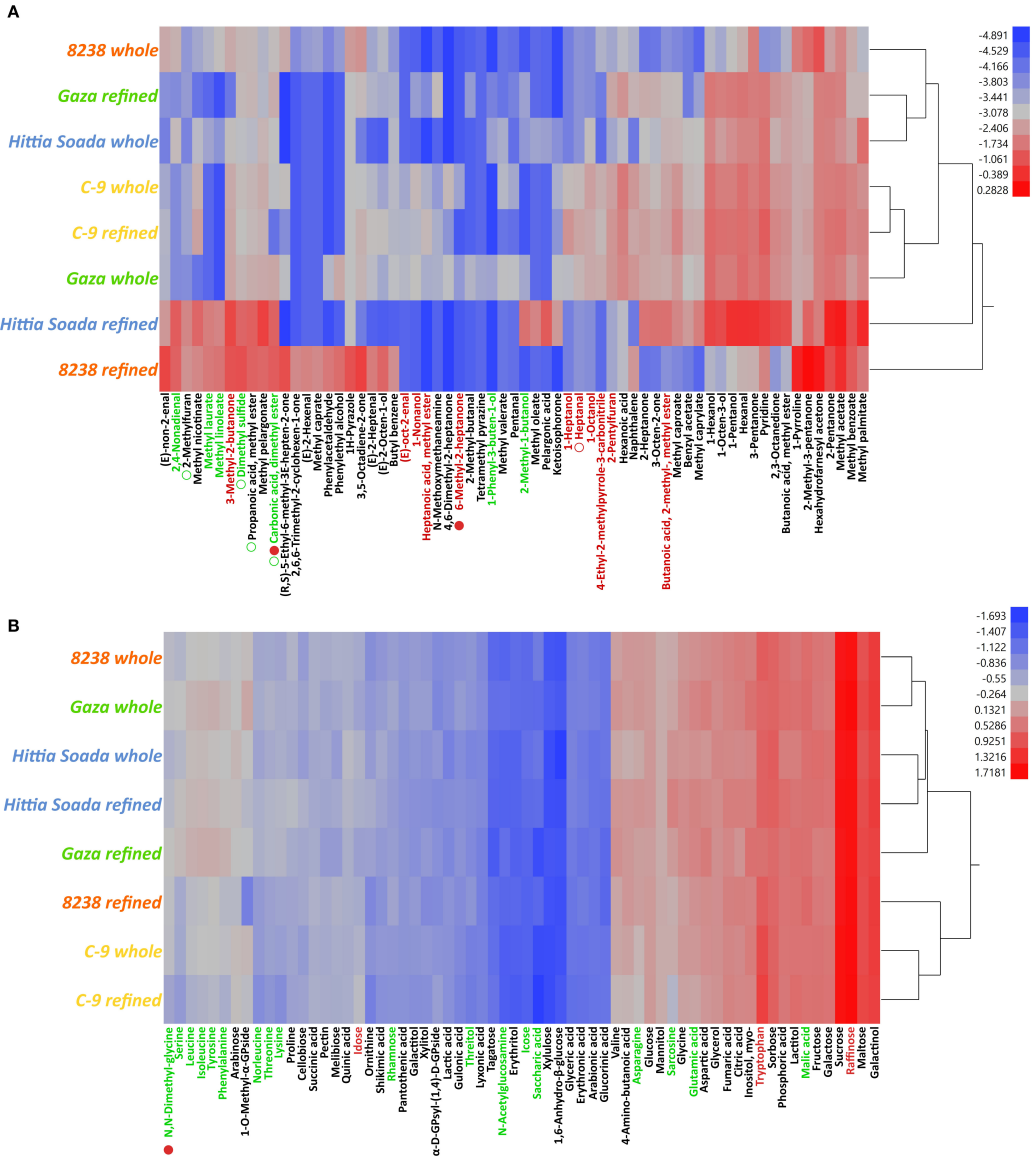
Aroma and taste compounds in *Triticum durum* flours (2019–2020). Figure present 2-way hierarchical clustering models (Ward's method, data is log_10_ transformed), of aroma **(A)** and taste-related compounds **(B)**. Metabolite concentrations are represented by an increasing color gradient from blue to red. Compounds marked in green font are significantly overrepresented in landraces; those marked in red font are overrepresented in the modern reference line. Full or empty bullets under the name denotes compounds that are either higher in whole or refined flours, respectively; bullet color represents whether this was evident in a landrace or a modern line, as mentioned previously. Dendogram and hierarchical clustering analysis were generated and visualized using JMP (version 13.2.0).

Aroma profiles of *T. durum* lines could be mainly discriminated by genotype (modern or landrace) followed by milling and their respective interaction (Figure 3A and Supplementary Table S1). In comparison to that of *T. aestivum*, the 2-way ANOVA model was far less robust due to the smaller set of lines and the fact that the representative landraces were exceptionally different from each other. The flour aroma of modern durum cv. C-9 was closely related to that of the landrace “Gaza” (and specifically to its whole flour) as compared with the two other lines “8238” and “Hittia Soada”. A handful of volatile compounds were overrepresented in durum landraces, including three fatty acid-related methyl esters (methyl laurate, methyl linoleate, and carbonic acid dimethyl ester). Landraces “8238” and “Hittia Soada” had strikingly different aroma profiles (Figure 3A, Supplementary Figure S3, Supplementary Table S1). “8238” refined flour, for example, exhibited two clusters of compounds that were uniquely over-accumulated, comprised of phenylpropanoids, terpenoids, and various fatty acid derivatives. A more detailed comparison between individual landraces and the modern reference C-9 can be found in Supplementary Table S2. The accumulation of taste-related compounds in *T. durum* was mainly driven by the genetic background, as evident by the primary separation of the modern C-9 reference from other lines (Figure 3B and Supplementary Table S1). This, similar to bread wheat, was due to significantly higher concentrations of amino acids (again, with the exception of tryptophan), as well as some sugars and organic acids in landrace flours. In contrast to bread wheat, this phenotype was evident regardless of milling type.

Evaluation of putative key odorants within compounds detected in flours was done by using an internal standard (*iso*butylbenzene) as a mean for assessing the individual odor activity value (OAV). Compounds with OAV > 1 (i.e., the ratio between their concentration and the known odor thresholds was over 1) were suspected as key odorants (Supplementary Table S2). Most of these were previously reported as aroma active compounds in either flour or breads (10, 11). The 23 candidate odor active compounds are noted in Supplementary Table S2. Out of these, ten were markedly higher in at least one landrace compared to the modern reference line: (E)-non-2-enal (fatty, green, cucumber, “Kandaharia” and “8238” whole and refined), 1-heptanol (green, “Juljuli” whole), 2,4-nonadienal (green, “Kandaharia”, “Hittia Soada”, and “8238” whole and refined), (E)-2-heptanal (green, “8238” refined), (E)-2-octen-1-ol (green, citrus, “8238” whole and refined), 3,5-octadien-2-one (fatty, fruity, “Lubnani Kisra”, “Palestinskaya”, “Juljuli”, “8238”, whole, and refined), benzyl acetate (floral, “Palestinskaya” whole and “Diar Alla” whole and refined), dimethyl disulfide (sulfurous, onion, “Karun” whole and refined), dimethyl sulfide (sulfurous, onion, “Karun” refined), and phenylacetaldehyde (floral, honey, “8238” refined) (Supplementary Table S2).

### 3.4. Sensorial evaluation of bread wheat and durum loaves

**Figure 4:**
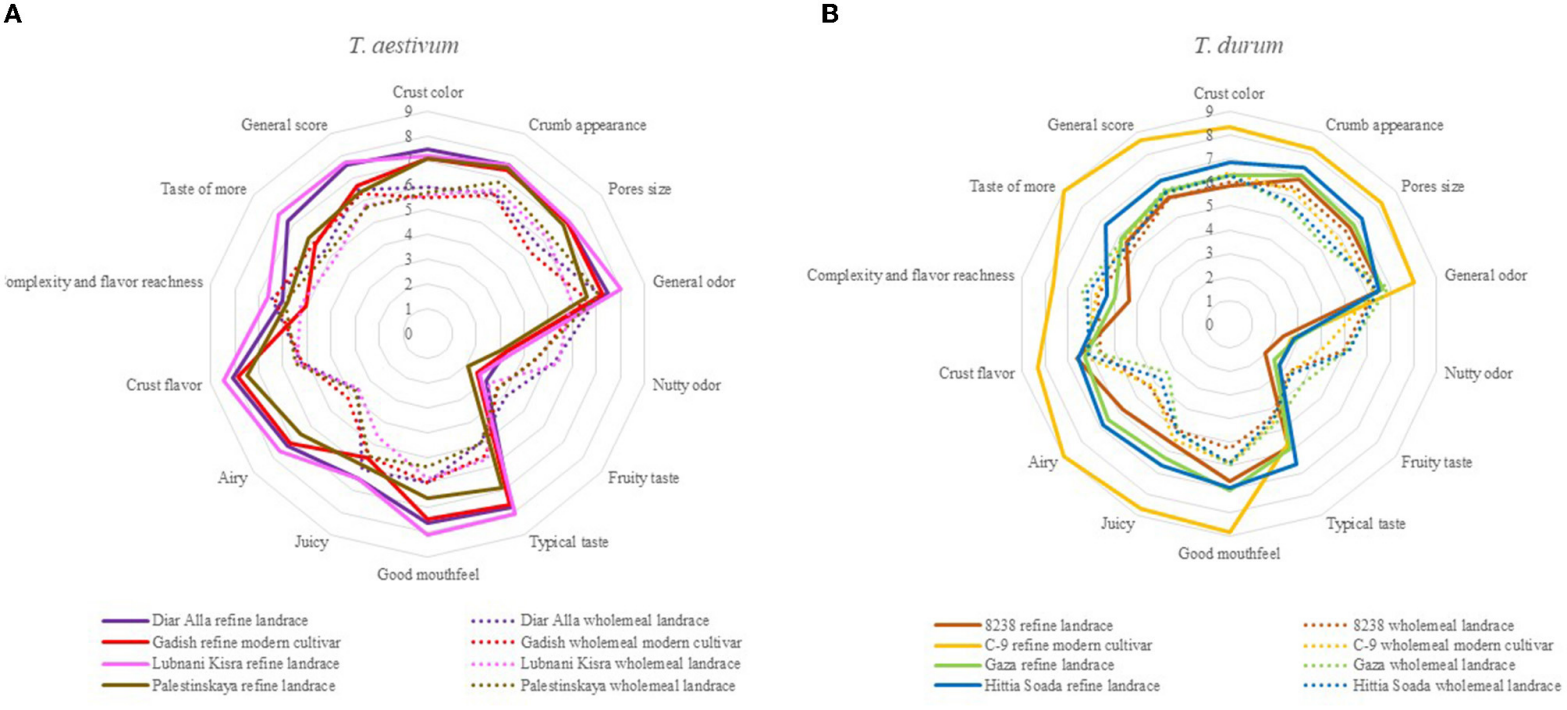
Sensorial panel preformed in 2021 for AACC protocol breads made of bread wheat **(A)** and durum wheat **(B)** for refined loaves (continuous line) and wholemeal loaves (dotted line). The sensorial panel was scored following a 1–10 scale where 1 represents the lowest score and 10 represents the highest score.

**Figure 5:**
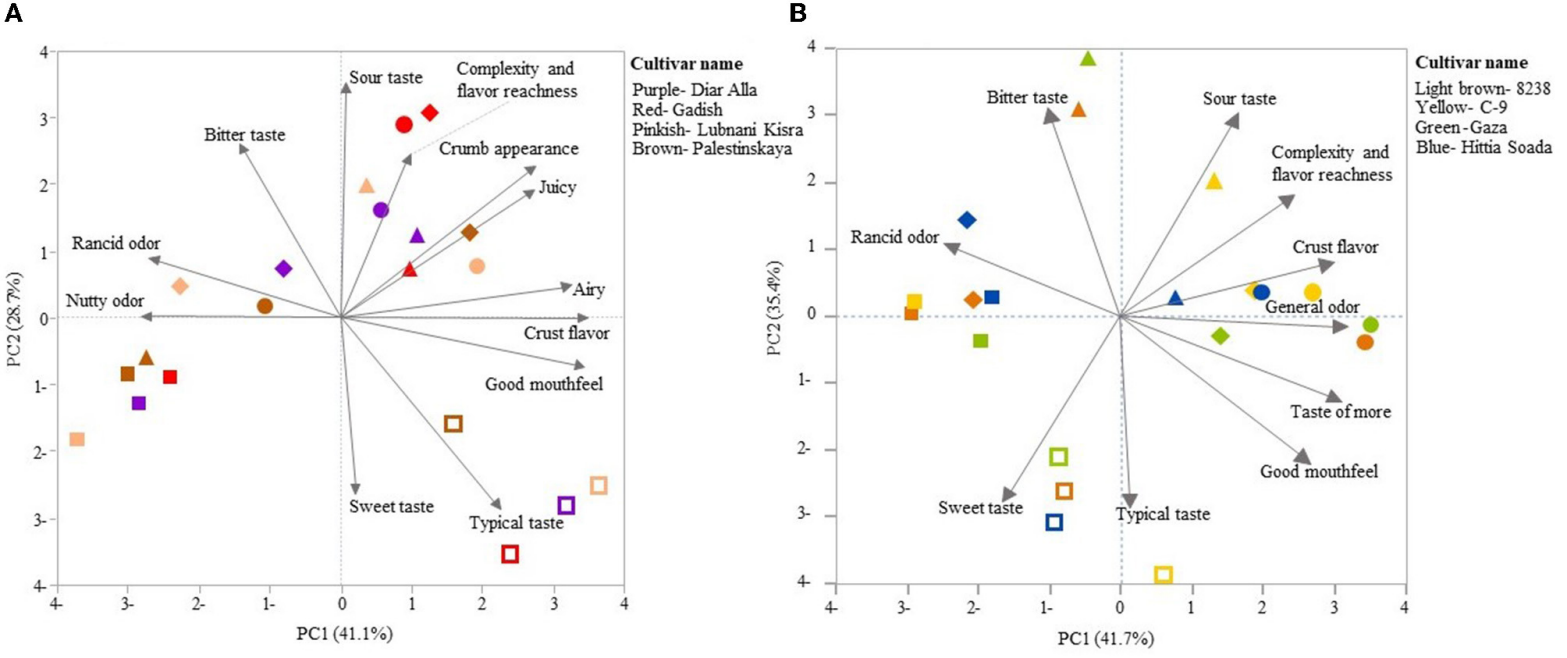
Sensorial panel performed in 2021 for artisan breads made of bread wheat **(A)** and durum wheat **(B)** by four artisan bakers (shapes) including refined loaves (hollow symbol) and wholemeal loaves (solid symbol). Sour dough bread (triangle, rhomb, circle); common protocol (square).

The sensorial panel was carried out twice over 2 years following the same protocol (2020 and 2021) for evaluation of the crust, crumb, taste, and odor of refined and wholemeal loaves. Data of the 2020 sensorial panel is presented in S1 for *T. aestivum* only, due to lack of a modern durum reference cultivar in the *T. durum* breads panel. Bread and durum wheat were evaluated separately (Figures 4A, B, respectively), where, in each assessment, loaves of three lines of landraces and one modern cultivar were examined, separating refined and wholemeal flour (Figures 4A, B) based on a common recipe for industrial yeast baking method. The first assessment examined *T. aestivum* lines: “Diar Alla”, “Lubnanai Kisra”, “Palestinskaya”, and cv. Gadish (Figure 4A). Panelists were served slices of bread with both crumbs and crusts. The spider web of sensory evaluation averages partially discriminates refined and wholemeal loaves with a clear preference toward refined breads (Figures 4A, B). Wholemeal loaves were scored high for “nutty odor” (as well as the *T. durum* panel, Figure 4B). Similarity between refined and wholemeal bread wheat loaves was expressed in “complexity and flavor richness” and “fruity flavor” (Figure 4A). Unlike the 1^st^ year of this study when the refined modern Gadish loaf was preferred in “general odor”, “typical flavor”, “airy”, and “crust flavor and color” (Supplementary Figure S1), in the 2^nd^ year, Gadish had no advantage over landraces in any of the parameters (neither whole or refined loaves), while refined loaves of “Lubnani Kisra” and “Diar Alla” led in “juicy”, “general score”, and “taste of more” (Figure 4A). The landrace “Palestinskaya” was less appreciated than Gadish in 2021 (Figure 4A) but the opposite trend was evident in 2020 only for refined loaves (Supplementary Figure S1). The wholemeal loaves had the same scores for “airy” and “crust flavor and appearance” but ranged wider for “crumb appearance“, “pore size”, and “general odor” (Figure 4A). The second assessment in 2021 examined loaves made of *T. durum* lines: “8238”, “Gaza”, “Hittia Soada”, and cv. C-9. Unlike bread wheat, the modern C-9 refined loaves were remarkably preferred by the panel of tasters (Figure 4B). Thereafter, “Hittia Soada”, “Gaza”, and “8238” were less valorized. There was less separation between wholemeal and refined flour loaves except for “nutty odor“, “typical taste”, “good mouthfeel“, and “airy”. Wholemeal durum loaves reached higher scores then refined loaves in “complexity and flavor richness” and “nutty odor“. When considering the recipes of three more artisanal bakers, principal components analysis (PCA) accounted for 69.8 and 77.1% of the sensorial evaluation variance in bread wheat and durum loaves, respectively (Figures 5A, B). In bread wheat loaves, PC1 accounted 41.1% of variance and clearly discriminated between refined and wholemeal loaves, followed by artisan bakers and genotype (modern or landrace), the less influential factor (Figure 5A). PC2 accumulated 28.7% of variance and separated the free-style sourdough loaves made by different artisanal bakers and the common protocol recipe based on industrial yeast. Refined loaves are loaded toward “typical flavor” and “sweet taste” while wholemeal-common recipe loaves are loaded with the “nutty odor” vector (Figure 5A). In durum wheat loaves, PC1 accounted for 41.7% of variance clearly dividing bakers' handprints with minor effect of the genotype, while PC2 explained 35.4% of variance separating refined and wholemeal loaves (Figure 5B).

### 3.5. The aroma profiles of wheat landrace breads

**Figure 6:**
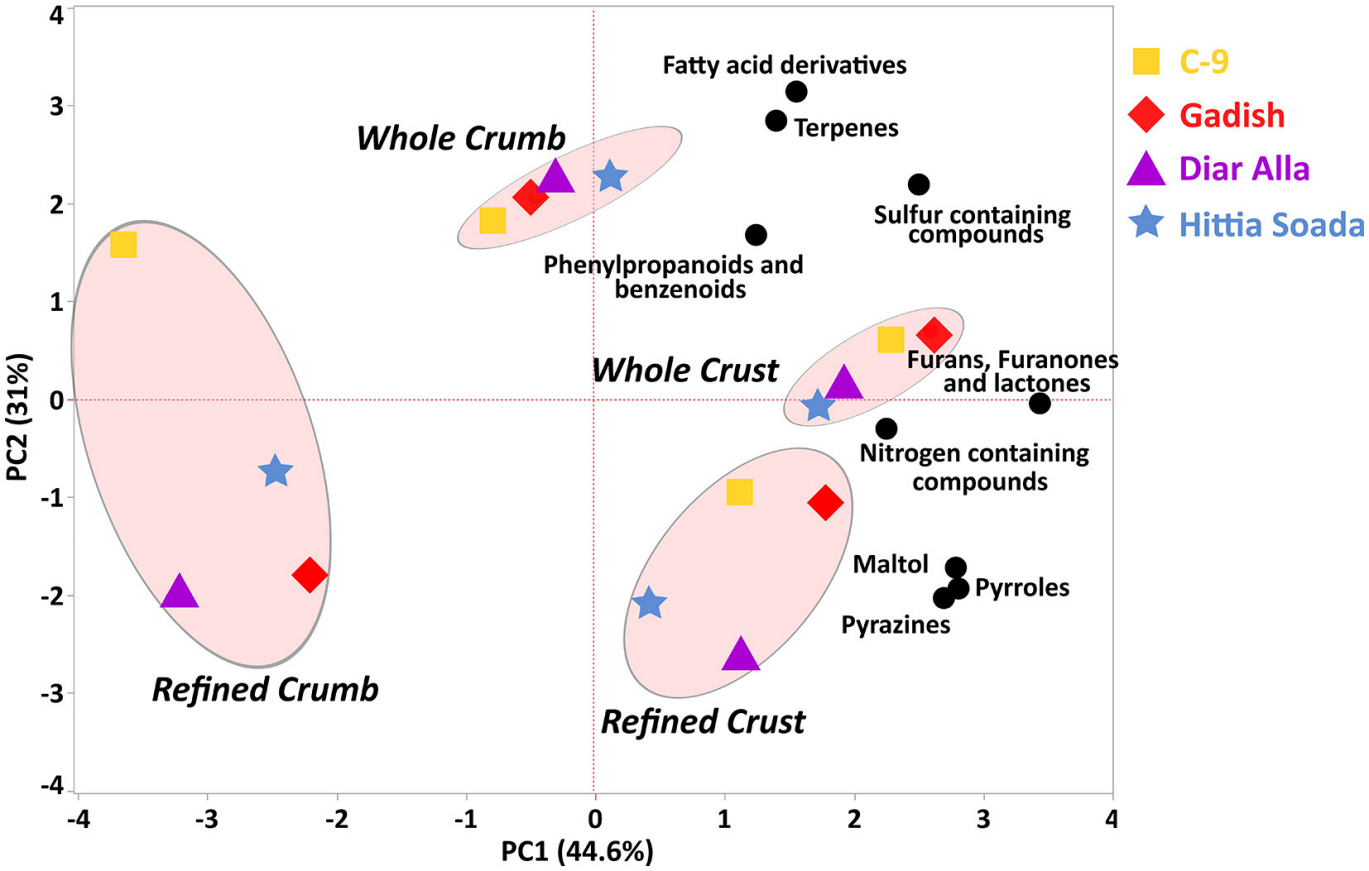
Principal components analysis of aroma classes detected in crumbs and crusts of loaves made using *T. aestivum* and *T. durum* flours, either landrace or modern. Values are sums of nine chemical classes found in breads; log_10_ transformed. Loading plot was generated and visualized using JMP (version 16.0).

Based on the sensory and analytical data, we chose one landrace of each species (“Diar Alla” and “Hittia Soada”) for further testing of the hypothesis that landraces may serve as a source for novel bread flavors. Loaves baked with landrace flour (crusts and crumbs from either whole or refined flours) were analyzed by HS-SPME-GCMS and compared with their corresponding modern references (“Diar Alla”/Gadish for *T. aestivum* and “Hittia Soada”/C-9 for *T. durum*). Since the volatile makeup of crumbs and crusts is known to differ considerably (13), we opted to analyze them separately. Overall, 205 volatile compounds were detected across all samples. These were classified into the six groups used for the flours, namely: lipid oxidation and fatty acid derivatives, nitrogen containing compounds, sulfur containing compounds, terpenes, phenylpropanoids, and furans (supplemented with the structurally related furanones and lactones arising from fermentation and baking). To these, three groups were added to reflect baking-originated Maillard and caramelization products: pyrazines, pyrroles, and pyranones (the latter containing only maltol) (Supplementary Figures S3, S4). Out of the 68 compounds detected in flours, ~68% were retained in baked breads (Supplementary Figure S4, Supplementary Tables S2, S3). Partitioning of individual volatiles show that whole flours tend to yield more diverse bread aromas. This is true for crumbs and crusts in bread wheat and only for crumbs in durum. Bread baked from whole flours are also more uniform across crumb and crust, while loaves from refined flours have less shared compounds between the two loaf sections, probably due to lower overall diversity (Supplementary Figure S5). The composition of bread aroma bouquets was mainly determined, as expected, by the sampling location, i.e., from crumb or crust. This was evident when analyzing the relationships between aroma profiles of all breads by PCA (Figure 6). The differentiation along PC1 (representing ~45% of variance) was mainly between crumbs/crusts, which directly corresponds to higher proportions of Maillard and caramelization products in crusts (Figure 6, Supplementary Table S3). A closer inspection of individual compounds across samples showed two main clusters of compounds over-accumulated in crusts, including alkylpyrazines, furans, pyrroles, maltol, and “Strecker aldehydes” (33), formed during later stages of the Maillard reaction (Supplementary Figure S4). Milling type was the secondary driving factor in determining bread aroma, as evidenced by a segregation across PC2 (representing ~31% of variance, Figure 6). Breads baked from whole flours were richer in terpenes, sulfur containing compounds, and fatty acid derivatives. Finally, samples were clustered according to their genetic background, i.e., modern or landrace, which was not evident in crumbs (Figure 6).

### 3.6. The aroma and taste profiles of specific lines within the panel

Next, we separately compared crumbs and crusts of breads prepared from landrace flours (refined or whole) to those baked from their corresponding modern reference lines. To that end, we analyzed both individual compounds as well as volatile classes, as reported in Supplementary Table S3. For *T. aestivum*, crusts of bread baked from whole flour of the landrace “Diar Alla” were richer in maltol, while having lower levels of furans, sulfur/nitrogen containing compounds, pyrazines, and terpenes. Crumbs of “Diar Alla” whole breads had lower levels of terpenes. Refined crusts of “Diar Alla” were markedly richer in maltol, furans (including furanones and lactones), and pyrroles, while having decreased levels of fatty acid derivatives, phenylpropanoids, pyrazines, and terpenes compared with the modern reference Gadish. The crumbs of loaves baked with Gadish refined flour were considerably more aromatic relative to those made from the landrace and manifested in higher levels of furans, maltol, pyrazines, and terpenes. For *T. durum* (Supplementary Table S3), pyrazine class was enriched in crusts of loaves prepared from whole flours of the “Hittia Soada” landrace. On the other hand, crusts of whole breads made from the modern reference C-9 had elevated levels of furans, phenylpropanoids, sulfur containing compounds, and terpenes. Crumbs of “Hittia Soada” whole breads were also richer in pyrazines, as well as maltol and fatty acid derivatives; their C-9 reference counterparts had higher concentrations of an assortment of unrelated compounds. Compared with C-9 crusts, the crusts of landrace durum refined breads had similarly higher levels of maltol and several pyrroles while simultaneously having decreased nitrogen containing compounds as well as phenylpropanoids. Finally, compared with the modern durum line, the corresponding crumbs of refined “Hittia Soada” loaves had elevated concentrations of maltol, pyrazines, and other nitrogen containing compounds, while having relatively low levels of phenylpropanoids (namely phenylethyl alcohol and benzoic acid methyl ester) as well as sulfur containing compounds.

It should be noted that only a handful of compounds retained their significantly elevated levels in landrace breads as well as in the flours used for baking (Supplementary Figure S4, Supplementary Tables S2, S3). The entire dataset of detected metabolites (*n* = 205) was cross-referenced to the current literature detailing odor active compounds in bread (10, 33). We found that our dataset contained 41 such compounds. Next, as with flours, we evaluated which compounds might have a critical contribution to bread aroma by roughly estimating their OAVs. Out of the 205 compounds, 32 had OAVs >1 (Supplementary Table S3) and an overlap of 17 compounds (53%) was noted between our OAV observations and the literature survey. A handful of compounds detected at OAV >1 were also significantly higher in breads baked from landrace flours, namely, 3-methyl-1-butanol (fermented, “Diar Alla” refined crust), octanal (aldehydic, waxy, citrus, “Diar Alla” refined crust), maltol (caramellic, “Diar Alla” refined crust), and 2-methyl-butanal (coacoa, “Diar Alla” whole crust). The crust of breads made with the refined flour of the modern cultivar Gadish was richer in these putative aroma active compounds: (E)-oct-2-enal (fatty), 1-octen-3-ol (earthy), 1-octen-3-one (earthy), (E)-2-decenal (fatty), 2-heptanone (cheesy), (E)-2-octen-1-ol (green), heptanal (green), hexanal (green), nonanal (aldehydic, fresh, waxy), phenylacetaldehyde (floral), 2,6-diethyl-pyrazine (nutty), and 2-ethyl-3,5-dimethyl-pyrazine (nutty). Durum reference C-9 was richer in 2-methyl-butanal (cocoa, whole crumb) and 2-methoxy-4-vinylphenol (spicy, whole crust).

**Figure 7:**
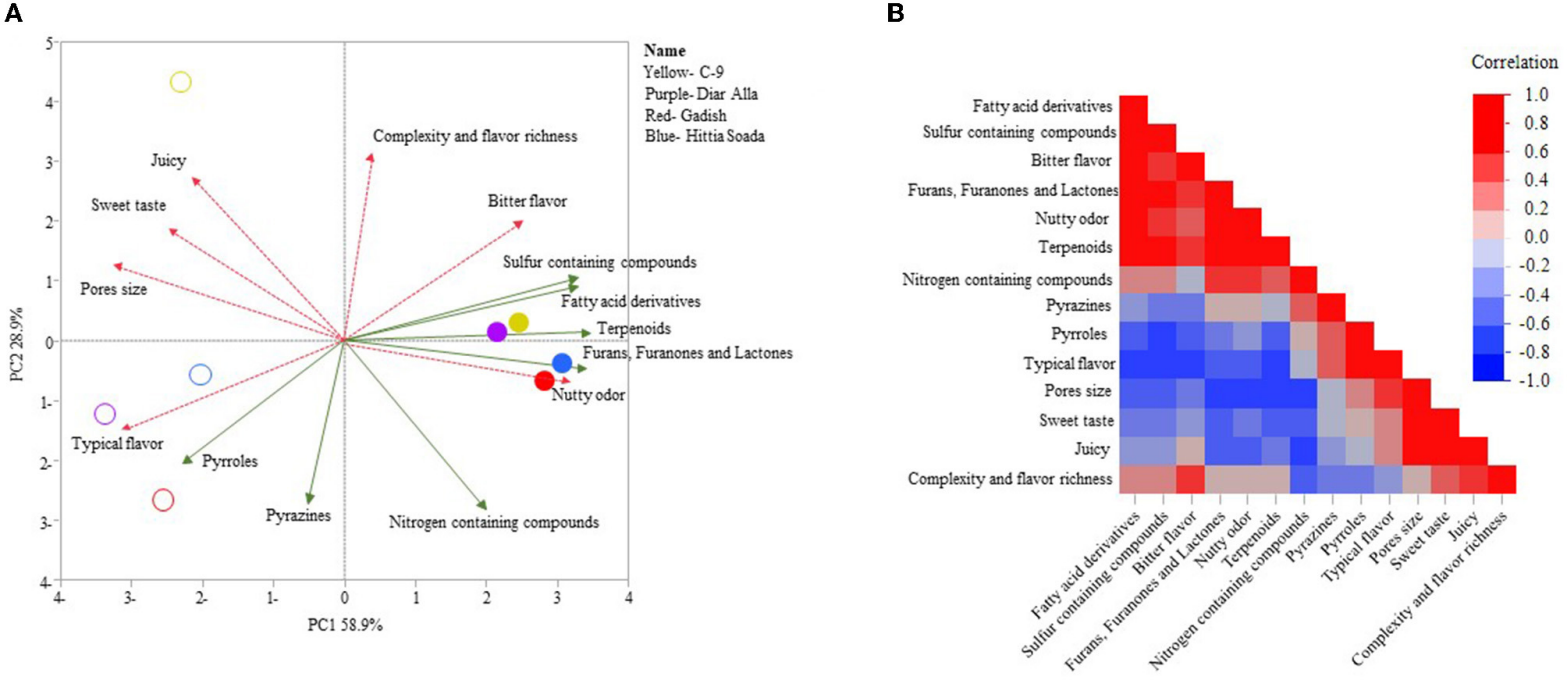
Principal components analysis and correlation of aroma compounds and sensorial score of the crumb of sensorial panel in 2021. **(A)** PCA of refined loaves crumb (hollow symbol) and wholemeal loaves crumb (solid symbols). Pink dashed vectors (sensorial score); green vector (aroma compounds). **(B)** Multi correlation between aroma compounds and sensorial evaluation.

**Figure 8:**
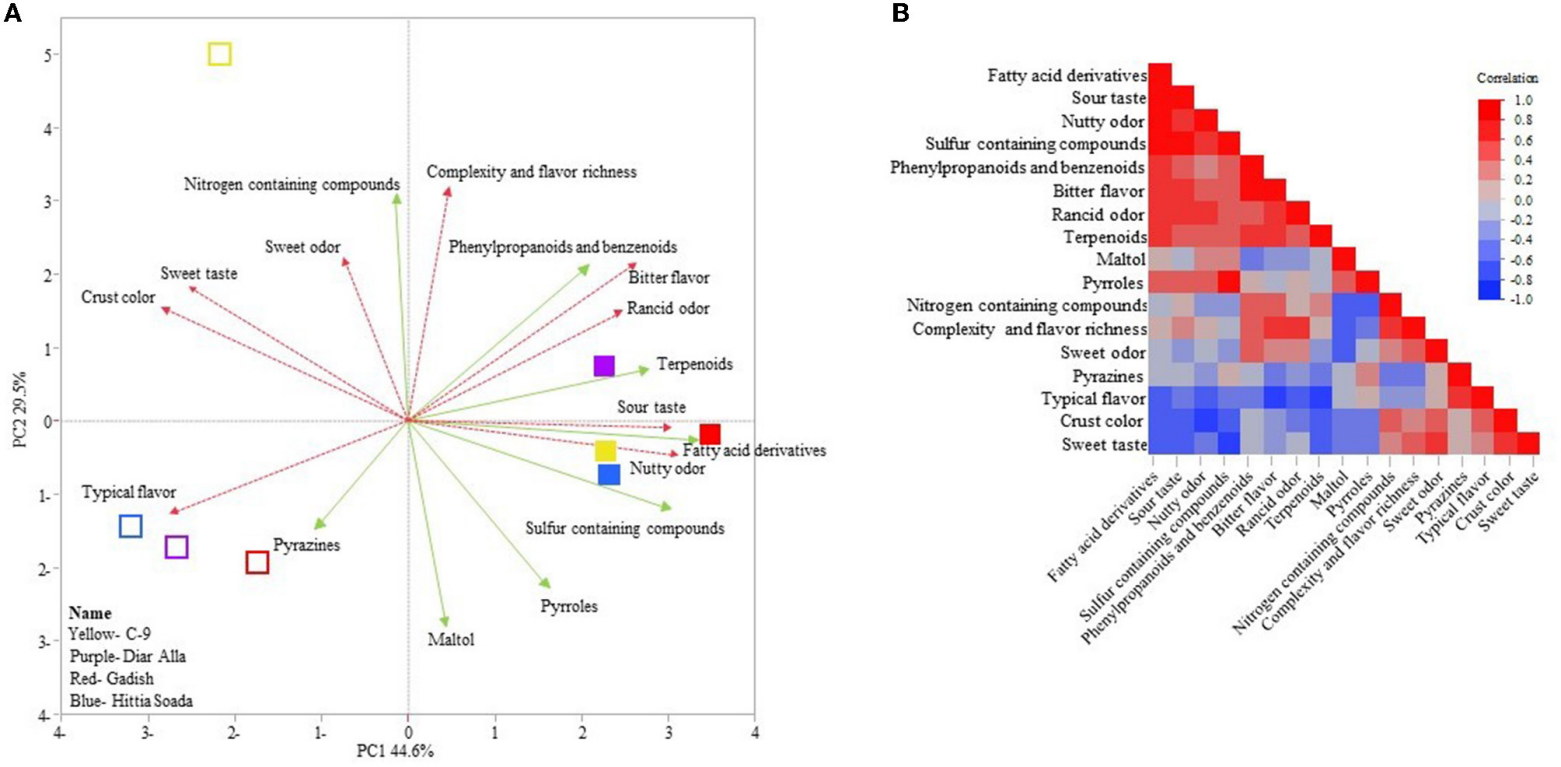
Principal components analysis and correlation of aroma compounds and sensorial score of the crust of sensorial panel in 2021. **(A)** PCA of refined loaves crust (hollow symbol) and wholemeal loaves crust (solid symbols). Pink dashed vectors (sensorial score); green vector (aroma compounds). **(B)** Multi-correlation between aroma compounds and sensorial evaluation.

To understand the correlation between sensorial evaluation and the presence of volatile compounds we conducted a separate analysis of the crumb and crust (Figures 7, 8), excluding hedonic parameters but keeping the following sensory descriptors: intensity of flavor, taste, odor, texture, and appearance. There was a strong correlation between sensorial evaluation and volatile compounds of the crumb or crust. PCA of the crumb accumulated 87.8% of variance, where PC1 accounted for 58.9% of the variation exposing the separation between refined and whole crumb (Figure 7A). PC2 represented 28.9% of the variation and mainly separated lines within each bread type (wholemeal or refined). This is especially evident for the refined C-9 crumb loaf separated (quarter iv) from the other three refined loaves (quarter iii). “Nutty odor” was associated with the crumb of wholemeal loaves and was correlated to fatty acid derivatives and furans, furanones and lactones (Figures 7A, B). Refined loaves with C-9 as exception were clearly defined by “typical flavor” that was correlated to pyrroles. In the crust PCA analysis, PC1, which explained 44.6% of the total 74.1% variance, again clearly discriminated refined from wholemeal loaves (Figure 8A). The wholemeal loaves' crust, loaded positively on PC1 were associated mostly with “sour taste” and “nutty odor” that were correlated with fatty acid derivatives and sulfur-containing compounds (Figures 8A, B). Refined crust breads were associated with “typical flavor” that was negatively correlated to terpenoids. Also in the crust, refined C-9 was isolated from refined “Hittia Sodada”, “Gadish”, and “Diar Alla” in the fourth quadrant, and was linked to “sweet taste” and “sweet odor”, crust color, and nitrogen-containing compounds (Figure 8A).

## 4. Discussion

The Israeli and Palestinian landraces (IPLR) collection was recently reported as a rich genetic resource for wheat crop improvement (19). Our main objective in this study was to assess the quality attributes and the flavor profiles of refined and wholemeal flours as well as the sensory evaluation of breads made of representative IPLRs in comparison to modern cultivars. By combining rheological, analytical, and sensory approaches, we assessed the whole and refined flour baking attributes and flavor profiles of three *T. aestivum* and three *T. durum* landraces, as well as those of modern commercial references. Our aim was to evaluate whether neglected Israeli wheat landraces have the potential to improve baking qualities and diversify the aroma and taste of bread.

### 4.1. Landrace vs. modern: quality and taste differences

The climatic conditions in wheat fields in Israel, typically representing east Mediterranean growing environments, are highly variable and fluctuate, causing substantial variability in wheat grain production and quality which is a matter of great concern for both producers and bakers (32–35). Grain quality parameters of the entire IPLR collection opens a hatch to the wide diversity of the landrace collection in key end-use quality attributes such as rheological properties, gluten matrix, kernel size, and pigment color (Figure 1, Table 1). The latter is reflected in the large number of durum landraces clustered on the yellowness vector (Supplementary Figure S2) which is in accordance with previous study highlighting 19 east Mediterranean durum landraces to have higher yellow pigment than a total of 165 durum landraces from the Mediterranean basin (36). The successful breeding in recent decades is reflected in this study with modern varieties yielding 2-fold compared with landraces following expected reduction in protein content (Table 1). This decrease is many times counterbalanced in modern germplasm by the rise in protein quality, also determining an increase in the amount of carbohydrates (37, 38). In the current study, modern cultivars had indeed higher wet gluten and GI compare to landraces, with the landrace “Diar Alla” as exception. However, it is worth noting that these parameters alone are not sufficient in determine bread making quality (32) without additional complimentary parameters (alveograph index and SDS, or bread volume). GI, especially, is somewhat problematic (32, 35), being environment-dependent. In our study this was clearly evident in the fluctuation values between seasons such as in the bread wheat “Lubnani Kisra” (GI = 71.9 and 17.4) and also in the durum “Gaza” (GI = 5.7 and 23.9). Dough rheology highlighted that the wheat landrace “Diar Alla” excelled in dough strength, tenacity, and development time in comparison with the modern cv. Gadish and the other two bread wheat landraces. The dough development time expresses the desired kneading time for optimal development of the dough and is measured by its stability (S) over time. The longer (S) duration, the stronger the flour and the pastry volume height (39). The extended DDT (17 and 21.9 min in 2020 and 2021, respectively) of “Diar Alla” in comparison to Gadish (5.9 and 7 min, respectively) might be appreciated by artisan bakers and be considered as an ingredient in a blend with modern cultivars to improve dough performance and the final bread characteristics. The modern durum C-9 showed a noticeable advantage in most baking-related qualities compared with the other durum lines (Table 1) and therefore might be used as a dual-purpose variety in terms of end-products. Along the consumer and bakers' chain, this durum variety was highly appreciated by both bakers and participants. This might expand the local bread industry perspective, which in general tends to avoid durum wheat except for leavened bread such as “Pane di Altamura” in Italy. Notably, the sensory evaluation did not differentiate between modern and landrace bread wheat loaves (wholemeal/refined) (Figure 4A). Only in breads baked from refined durum flours was C-9 markedly favored over the other lines (Figure 4B). Interestingly when comparing the preference of the panelist for loaves made by the artisan bakers, it was found that tasters' preferences were mostly derived from the flour type factor and to a lesser extent the baking style. Here, too, there was no discernible separation between modern cultivars and landraces (Figures 5A, B).

### 4.2. Are landrace and modern flour aromas different?

By way of analytical chemistry, and more specifically HS-SPME-GCMS, we detected 68 compounds in the aroma bouquets of whole or refined flours, a result reported in Supplementary Figure S4. Our current results are comparable with previous works that have attempted similar analyses (Supplementary Table S2) (11, 23, 40). The genetic background component (i.e., landrace or modern cultivar) was evidently the major factor discriminating the dataset (Figures 2, 3, Supplementary Table S1). Furans, terpenes, and phenylpropanoids, as well as fatty acid-related methyl esters, were accumulated in higher levels in *T. aestivum* landraces. *T. durum* lines had fewer overlapping components in terms of aroma, implying that this subset is probably more diverse (Figures 2, 3). The interaction between milling and genotype was mainly noted for the modern varieties, since the enrichment of taste- and aroma-related metabolites in refined flours and taste compounds in whole flours was almost exclusively observed in modern lines and far less for landraces, which exhibited uniformity between milling types (Figure 2, Supplementary Table S4). It is plausible to assume that this is due to modern breeding targeting refined flour qualities, i.e., heavily focusing on endosperm quality at the expanse of those of bran and germ. Overall, these findings are corroborated by previous studies comparing the flavor makeups of modern and landrace germplasm (1, 24, 26). For example, Ficco et al. (26) conducted a multi-factorial study into the contributions of genotype, milling, leavening agents, and baking method on baking quality and sensory attributes (including aroma composition) of a durum landrace. The authors report that genotype (landrace or modern) had the highest contribution in determining levels of flour aroma (including alcohols and terpenes, but less so for aldehydes), followed by milling and the interaction between the two (note that bread aroma was differently affected by these factors). Similarly, Starr et al. (23), demonstrated that European bread wheat lines present a different pattern of aroma compositions based on them being ancient or modern, manifested in higher levels of esters, alcohols, and furans in landraces. Together with our data, it seems that the line identity plays a key factor in shaping flour aroma, which may offer some attractive targets for breeding. This might be relevant for specific volatiles that are considered key odorants, since the biosynthesis of these end-products is executed by enzymes encoded by single genes or gene-clusters (41–43). Studies in other cereal models such as maize and rice described the isolation and characterization of such genes (44, 45). A genome-wide association study using an appropriate population pool might provide the first steps toward breeding superior-tasting flours, with the data presented here and in other works to flag potential compounds. In any case, it is clear that the flour aroma of wheat landraces differs from that of modern lines and may very well be used for diversifying wheat aroma. Recent studies have emphasized the beneficial characteristics that ensure superior performance of bread made of durum wheat (46) for its high protein content and strong gluten. Mastrangelo and Cattivelli (47) reported well-described genes for qualitative traits. The authors suggested developing wheat lines with a durum or bread quality make-up in either a tetraploid or hexaploid genetic background to produce pasta and bread, respectively, especially in the framework of local traditions.

Monitoring the levels of aroma compounds is crucial in understanding food quality, but not all compounds necessarily contribute to the actual perceivable flavor. We assessed which of the metabolites detected in flours could potentially be considered as key odorants by using an internal standard and found 23 such compounds, comparable to the figures reported in previous studies (11, 13, 26). Out of this subset, ten compounds were found to be at significantly higher levels in landrace flour compared to the modern reference lines. This result corroborates our findings showing that landrace flours are a viable source for new aroma compositions, most likely with green, fresh, fatty, and floral notes (Supplementary Table S2). We acknowledge that our approach is rather descriptive and preliminary, and that our data requires a more comprehensive analysis to validate these assessments. There are several methods for determining aroma active components of food, such as calculating OAVs with authentic standards, performing aroma extract dilution analysis for extracts or specific compounds, using GC-olfactometry, or a combination of these (48, 49). Future investigations will necessitate the use of these methods together with sensory approaches for in-depth analysis of wheat landrace aroma.

### 4.3. Wheat landrace flour flavor potential

We complemented the investigation into the flavor potentials of landraces by targeting and measuring the levels of taste-imparting compounds, including sugars, organic acids, and amino acids. The resulting set of metabolites was comparable, at least qualitatively, to previous studies (Supplementary Table S2) (40). To the best of our knowledge, this is the first study showing that wheat landraces contain higher levels of taste-related compounds compared to modern lines. For both species, this was evident in higher levels of several metabolite classes including sugars important for baking like glucose, sucrose, and fructose, but most strikingly of free amino acids: asparagine, glutamic acid, isoleucine, leucine, lysine, N-acetylglucosamine, phenylalanine, serine, threonine, and tyrosine (Figures 2, 3). In plants, amino acid metabolism first utilizes substrates from glycolysis (aromatic and branched-chain amino acids, alanine, glycine, cysteine, and serine), citric acid cycle (asparagine, lysine, threonine, methionine, isoleucine, glutamic acid, glutamine, proline, and arginine), and the pentose phosphate pathway (histidine and aromatic amino acids). Without further molecular and genetic research, it will be difficult to explain the over-accumulation of free amino acids in landrace flours. Regardless, this finding may suggest superior flavor for landrace wheats, as well as the capacity to utilize these metabolites in forming other flavor-related compounds in fermentation, baking, or cooking. One notable exception was observed for the amino acid tryptophan, which was consistently higher in modern lines (Figure 3B). It should be noted that tryptophan serves as a precursor for several secondary metabolites, including defense compounds (e.g., indole glucosinolates), hormones involved in pathogen resistance (auxin), and is itself a product of indole, a metabolite implicated in plant defense (50–52). Elevated levels of this amino acid in modern lines could potentially represent evidence for guided breeding that would favor pest-resistance. Interestingly, studies of seed composition of wild and domesticated chickpea have found that the latter contain markedly higher tryptophan levels (53). It was proposed that since tryptophan is the precursor for serotonin, a neurotransmitter associated with positive effects in humans, this might represent an ancient selection trend that resulted in domestic cultivars with increased levels of this amino acid (54).

### 4.4. Is landrace bread aroma significantly different from modern bread?

To test whether landrace breads have different flavor profiles compared to those baked from grains of reference modern cultivars, loaves of each of the four lines were prepared from whole or refined flours of one landrace and its reference modern cultivar from each species. Since crumbs and crusts were clearly the main factor driving the variability between samples (Figure 6), we opted to analyze samples arising from these parts independently (Supplementary Figure S4). We detected 205 aroma compounds across all samples, classified into nine groups. Since some aldehydes and alcohols may arise from yeast amino acid metabolism (Ehrlich pathway) Maillard reaction (Strecker aldehydes) and grain-based amino acid or fatty acid catabolism, we had no definitive way of determining their source and so kept the original classification. Around 68% (46/68) of the compounds detected in flours retained in breads after baking, but only nine compounds were measured at a significantly higher level in both the landrace flours as well as its corresponding bread compared with the modern reference cultivar. Aroma compounds do not always persist at the same levels in cooked food, and specifically in bread, due to their volatility, dilution effect, and addition or reduction due to yeast/bacteria activity (9–13, 17). The observation that only a fraction of compounds presents the same trend suggests that flour aroma may not be the optimal proxy for bread flavor. However, we propose that using landraces for other products, e.g., pasta, cookies, and couscous could rely directly on the flour aroma analysis for the purpose of designing and predicting the quality of the end-product.

Loaves baked from the landraces “Diar Alla” and to a lesser extent “Hittia Soada” presented a markedly different aroma from the control loaves prepared from modern flours, both in terms of overall compositions and individual compounds, including classes such as pyranones, pyrazines, furans, and pyrroles (maltol), some of which were documented as key odorants of bread and important for consumer acceptance. Modern cultivars, on the other hand, were consistently richer in terpenes and phenylpropanoids (Figures 2A, 3A, Supplementary Table S3, Supplementary Figure S4). An estimation of aroma active compounds yielded 32 compounds with putative OAVs >1, highlighting them as possible key odorants, with a handful of these enriched in landraces, imparting fermented, aldehydic, caramel, and cocoa notes. Whole and refined crusts of “Diar Alla” and all samples of “Hittia Soada” (except whole crust) had higher proportions of maltol, one of the compounds most associated with the aroma of freshly baked bread (48). Maltol is formed either through the caramelization of disaccharides or through the Maillard reaction intermediate 1-deoxysone. “Diar Alla” flours indeed contain increased levels of some disaccharides (e.g., sucrose and maltose) compared to Gadish, which could account for the elevated maltol formation in bread. However, “Hittia Soada” flours do not contain more disaccharides than C-9 but still accumulate more maltol, which might suggest that levels of free sugars do not correlate with those of their end-products after baking. Similarly, “Hittia Soada” breads contained higher proportions of pyrazines and pyrroles, which could theoretically be explained by associating it with the levels of free amino acids and reducing sugars in the flours. Flours of both landraces indeed had higher concentrations of free amino acids yet we did not observe a similar pattern of Maillard and caramelization product formation in “Diar Alla” (except for refined crusts). This informs us that non-bound, free amino acids and sugars could be important for flavor but are probably secondary in terms of serving as precursors for baking-related products. A potential source for these baking-related products is starch and protein degradation during autolysis and fermentation (55). “Hittia Soada” kernels contain more total protein compared to the C-9 cultivar and exhibit slightly higher thousand kernel weight (TKW), a proxy for starch content. “Diar Alla” has similar protein content but higher TKW values to those of Gadish (Table 1). However, we do not have data regarding the endogenous proteolytic and starch degrading enzymatic activity nor did we measure basic taste compounds in the dough. Further rheological and analytical measurements during fermentation as well as better accuracy in quantifying Maillard reaction compounds and intermediates would improve our knowledge about the relationship between precursors and end-products of landrace bread flavor.

### 4.5. Can landraces be bred intentionally for wholemeal products to improve the current artisanal market?

The transition from a wholemeal flour-based market to refined flour is a rather recent phenomenon associated with the industrial introduction of steel rollers mills. The automated production of fine white flour was based on a loss of benefits attributed to the bran and embryo section of the grain, with wide and dramatic implications on human nutrition and health [see comprehensive review of Dror et al. (56)]. Another consequence was a turnover in wheat breeder's goals in a way that for the last 150 years focused on refined flour quality, targeting exclusively endosperm quality. Modern millers and breeders' prioritize solid bran that is easily removed in the milling process, inevitably ignoring wholemeal flour qualities and the possible benefits of the bran and embryo. As wholemeal products are becoming increasingly popular for consumers, including the understanding of its health benefits (56), this might imply that improving wholemeal product taste using elite breeding material has a strong genetic constraint simply because bran and germ quality parameters were not being studied, evaluated, and consequently selected for. This genetic bottleneck might also suggest that a significant portion of bran and germ trait diversity was rapidly lost from modern germplasm. Wholemeal bread is considered less appreciated by consumers (57), as also reflected by our results (Figure 4). In that sense, breeders should consider landraces for wholemeal breeding to “restore a crown to its former glory”. As wheat landraces were used for wholemeal flour-based food in the Mediterranean cuisine for over ten millennia, they might harbor untapped reservoir for tastes and aromatic ingredients of wholemeal products. The artisan bakers' recipes together with sourdough-based baking created a spectrum of flavors that received a very diverse evaluation in both durum and bread wheat loaves. One of the interesting results in this study was the positive evaluation of the panelists for wholemeal sourdough loaves (both durum and bread wheat) compared to commercial-yeast loaves (Figures 5A, B) that received a uniform valorization for all the lines. This was especially so for durum wholemeal yeast loaves that were scored with “rancid smell”, including one type of free hand “Hittia Soada” loaf (Figure 5B). The positive evaluation of wholemeal sourdough breads was highly correlated to some of the aroma compounds (Figures 7, 8). “Diar Alla” wholemeal loaf was characterized with “fruity taste” in its crumb and crust, which was highly correlated to sulfur-containing compounds, terpenoids (Figure 7B), and fatty acid derivatives (Figure 8B), some of which indeed confer these types of sensory descriptors. The correlation between sensorial profile and aroma compounds should be further examined as a pre-breeding tool that might save the costly sensorial descriptive analysis (58) and correctly predict the desired end flavor (1) by targeting the aroma compounds that were significantly correlated to panelists preferences. The IPLR collection preserves 728 durum and 173 bread wheat accessions of which only six went through an in-depth characterization of flavor tests and aromatic profiling in this study. The diversity of the IPLR collection in grain quality aroma and taste is far from being fully reflected in its entirety in this subset. Our results can only provide a glance on the potential of wheat landraces justifying further exploration of this exotic genetic resource for the local and global bread industry.

This study follows up a previous study on the agronomic performance and adaptation of landraces in the Mediterranean environment (20). Our current findings indicate that landraces have the potential to improve flavor and aroma in wholemeal bread, even more so when it is made of sourdough over industrial yeast-based bread; then, the sensory appreciation is scattered over a wider range. For artisan bakers, the research findings might give an added value for differentiating boutique bakeries by applying the unique characteristics of selected landraces such as “Diar Alla” to improve the taste and aroma of wholemeal bread to improve dough management with interesting rheology advantages, or to incorporate them as additional flour in the blend together with elite varieties. Our results stressing the interaction between sensory evaluation scores and volatile compounds, might be the first step toward incorporating aroma selection parameters in wheat pre-breeding programs. Highlighting the aroma compounds that are associated with positive preferences should be further examined as an auxiliary tool for the quality traits of possible large scale genotype selection. The fact that analytical techniques are becoming faster, cheaper, and more adapted to field conditions, especially when targeting a predefined set of metabolites, enhancing its feasibility as a pre-breeding tool in modern breeding programs. These chemical-sensory associations are already pointing toward a unique flavor for landrace breads; however, the full composition of their aroma profiles may also play an important role. This is because of complex interactions between sets of volatile compounds, yielding masking, and synergistic effects. While some progress has been made in understanding the types of consequences of volatile interactions, this is mostly done in pairwise comparisons rather than with multiple compounds (59). This field is still at its infancy and new techniques and approaches will be needed to understand these complex interactions (60). Overall, our results can support the revival of local bread supply chains following a comprehensive understanding of the varietal performance of the local landraces.

## Data availability statement

The original contributions presented in the study are included in the article/Supplementary material, further inquiries can be directed to the corresponding author.

## Author contributions

SF, AC, DJB, VT, BG, and RBD conceived and designed this study. SF and AC coordinated the study and drafted the manuscript. SF, AC, DJB, KN, DD, YS, MB, MI, HG, CL, and JC undertook data collection. SF and AC conducted the analysis and modeling with DJB, VT, BG, and RBD providing critical comments. DJB, VT, MI, SA, and RBD provided revision of the manuscript. All authors have read and agreed to the published version of the manuscript.

## Supplementary material

Supplementary Figure S1 Sensorial panel in 2020 for common protocol breads made of bread wheat for refined loaves (continuous line) and wholemeal loaves (dotted line).

Supplementary Figure S2 Principal component analysis of grain quality compounds of landraces from the IPLR collection and modern cultivars. IPLR collection (gray symbol); modern cultivars (orange symbol); subset landraces (black symbol); hexaploid accessions (round dot); tetraploid accessions (triangle). Grain protein content (PRO), thousand kernel weight (TKW), yellowness, sodium dodecyl sulfate sedimentation volume (SDS), gluten index (GI), and wet gluten (GLU). Biplot vectors are trait factors loading for PC1 and PC2.

Supplementary Figure S3 Aroma class composition of bread wheat and durum flours (2019–2020). The compositions of aroma compounds in flours are presented as the relative proportion of each of the six main groups of volatiles. **(A)** Whole and **(B)** refined flours in lines of *T. aestivum*; **(C)** the corresponding flours of *T. durum*.

Supplementary Figure S4 Aroma and taste compounds in crumbs/crusts of breads prepared from landrace flours (2019–2020). Presented are 2-way hierarchical clustering models (Ward's method, data is log_10_ transformed, *n* = 3), using aroma compounds detected in crumbs and crusts of breads prepared from landrace lines (“Diar alla”/“Hittia soada”) or modern reference cultivars (“Gadish”/“C-9”). Metabolite concentrations are represented by an increasing color gradient from blue to red. Classes of aroma compounds are marked red; metabolites that were present in flours are marked yellow; those marked with green stars retained their increased levels in breads as well as in flours. Dendogram and hierarchical clustering analysis were generated and visualized using JMP (version 14.0.0).

Supplementary Figure S5 Partitioning of volatile compounds across breads used in this work (2019–2020). Presented are Venn diagrams representing the partitioning of volatile compounds detected in crumbs and crusts from whole and refined bread wheat and durum lines: “Diar alla” **(A)**, “Gadish” **(B)**, “Hittia soada” **(C)** and “C-9” **(D)**. Diagrams were prepared using the online University of Gent Venn diagram tool (www.bioinformatics.psb.ugent.be/webtools/Venn).

Supplementary Figure S6 Aroma class composition of bread wheat and durum breads (2019–2020). The compositions of aroma compounds are presented as the relative proportion of each of the nine main groups of volatiles detected in crumbs or crusts of breads prepared from whole and refined flours of modern and landrace lines, *n* = 3. Composition of **(A)** crusts and **(B)** crumbs of breads prepared from whole flours; **(C, D)** are the corresponding compositions of breads prepared from refined flours.

Supplementary Table S1 Two-Way ANOVA model results for factors shaping flour aroma (2019–2020). Presented are scores for whole models as well as individual significant compounds (^*^*p* < 0.05, DF = 1) of bread wheat and durum whole and refined flours. For each species, comparisons are divided into breeding (i.e., line identity; modern or landrace), milling and their subsequent interactions for either aroma or taste compounds. Statistical analysis was executed using JMP (version 14.0.0).

Supplementary Table S2 The aroma and taste of flours tested in this work (2019–2020), including comparison between bread wheat and durum landrace and modern cultivars (*n* = 3–4, means ± SE), odor descriptors (mined from www.thegoodscentscompany.com), odor thresholds (in μg/g), and odor activity values (OAVs, values >1 are marked with “O”). Aroma concentrations are expressed as μg/gr, taste compound values are expressed as the ratio between the peak area of the compound and that of the internal standard (ribitol). Refined or whole landrace flours were compared to those of modern ones using Dunnet's multiple comparison test results (compare with control). Significant comparisons are marked with an asterisk (^*^*p* < 0.05). Statistical analysis was executed using “R” package “DescTools”.

Supplementary Table S3 The aroma of breads tested in this work (2019-2020), including comparison between breads made with flours of bread wheat and durum landrace and modern cultivars (crumbs and crusts, *n* = 3, means ± SE), odor descriptors (mined from www.thegoodscentscompany.com), odor thresholds (in μg/g), and odor activity values (OAVs, values >1 are marked with “O”). Whole/refined crumbs and crusts were compared between landrace and modern lines using Student's *t-*test. Significant comparisons are marked with an asterisk (^*^*p* < 0.05). Statistical analysis executed using “R” package “DescTools”.
