# Supplementary figures and images for: New flavors from old wheats: exploring the aroma profiles and sensory attributes of local Mediterranean wheat landraces
Source: Front Nutr. 2023 May 18;10:1059078. doi: 10.3389/fnut.2023.1059078 (PMC10234510; doi:10.3389/fnut.2023.1059078)

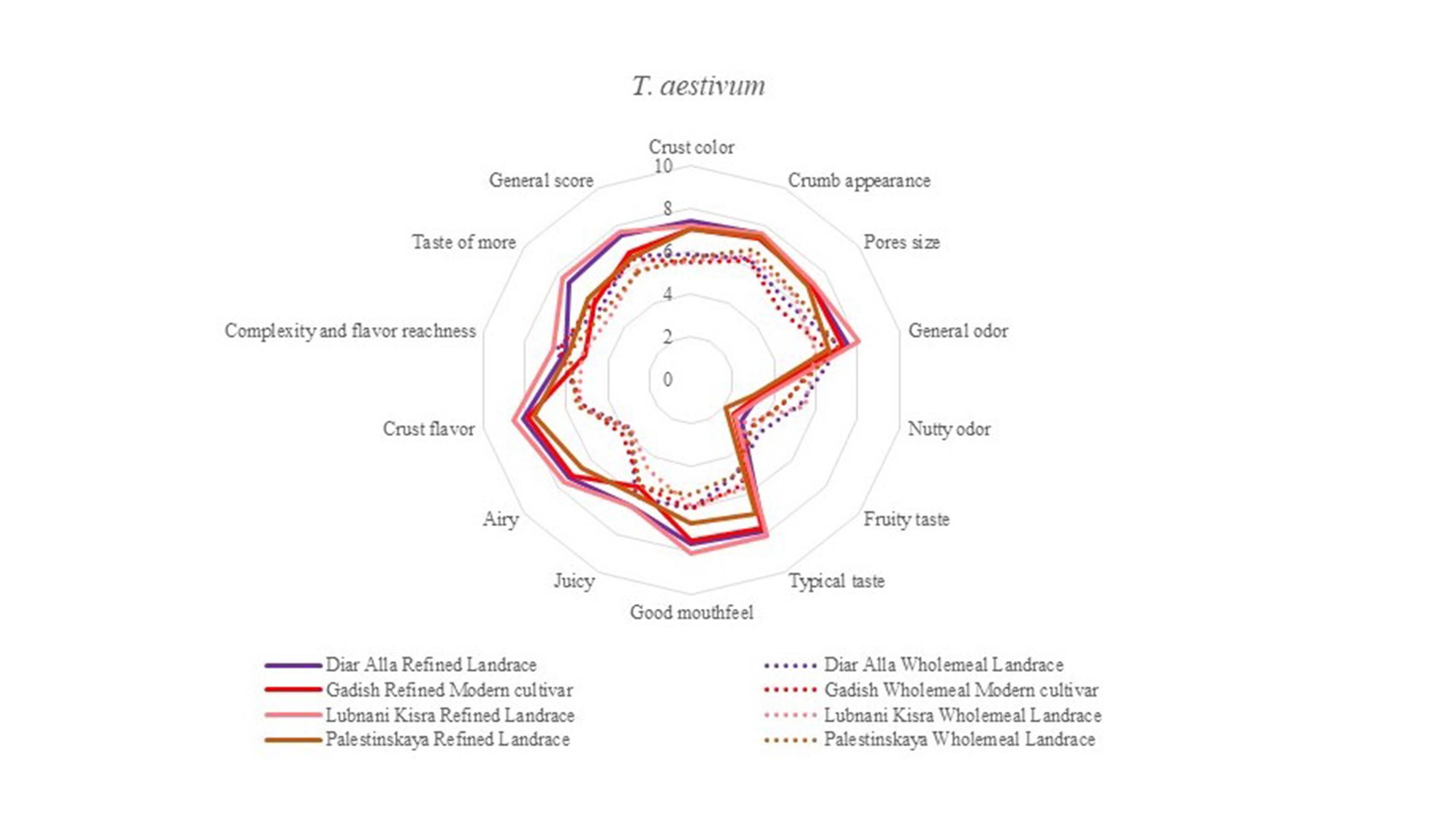

Supplement: Supplementary Figure S1 — Sensorial panel in 2020 for common protocol breads made of bread wheat for refined loaves (continuous line) and wholemeal loaves (dotted line). [file Image_1.JPEG]

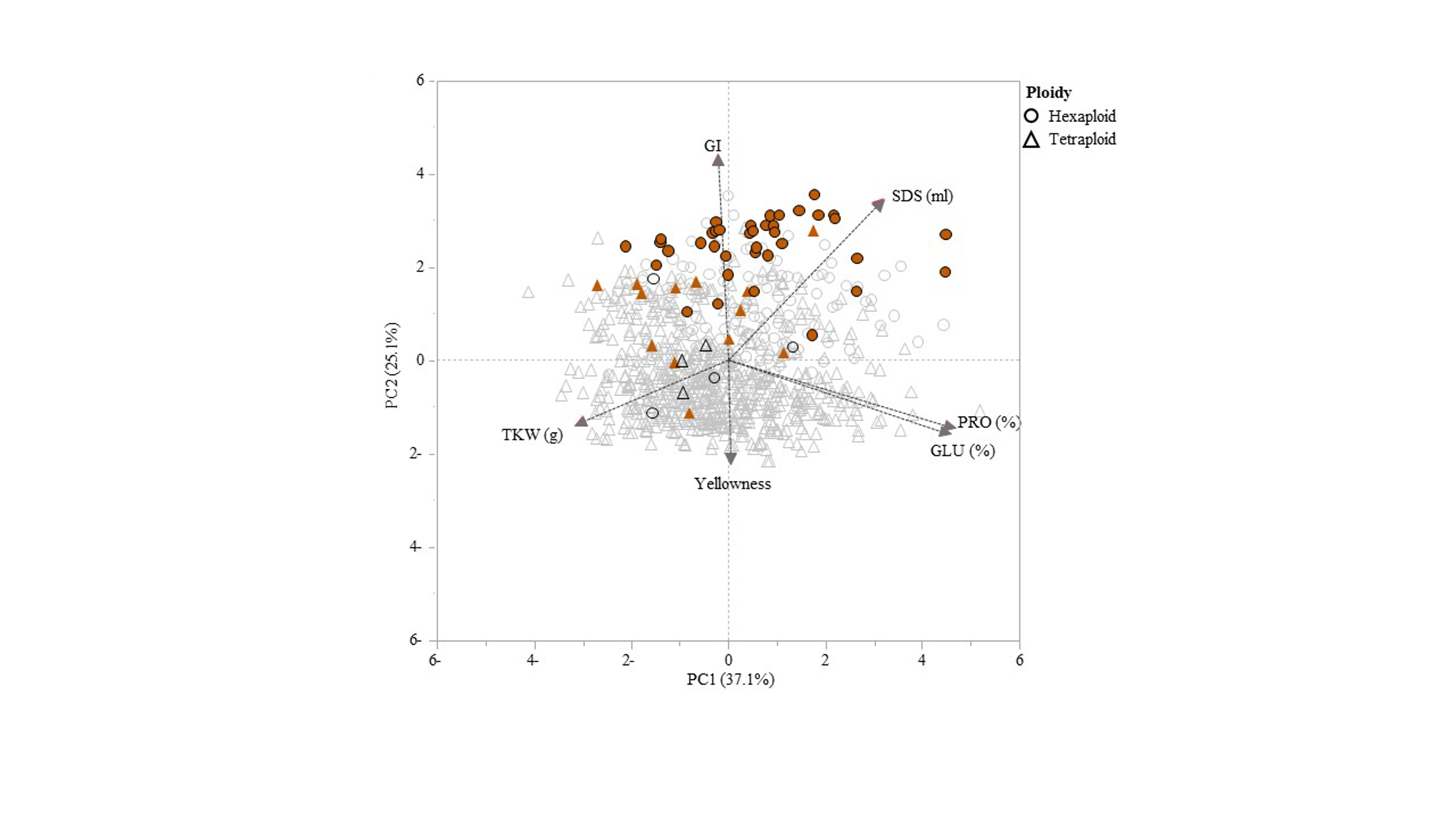

Supplement: Supplementary Figure S2 — Principal component analysis of grain quality compounds of landraces from the IPLR collection and modern cultivars. IPLR collection (gray symbol); modern cultivars (orange symbol); subset landraces (black symbol); hexaploid accessions (round dot); tetraploid accessions (triangle). Grain protein content (PRO), thousand kernel weight (TKW), yellowness, sodium dodecyl sulfate sedimentation volume (SDS), gluten index (GI), and wet gluten (GLU). Biplot vectors are trait factors loading for PC1 and PC2. [file Image_2.JPEG]

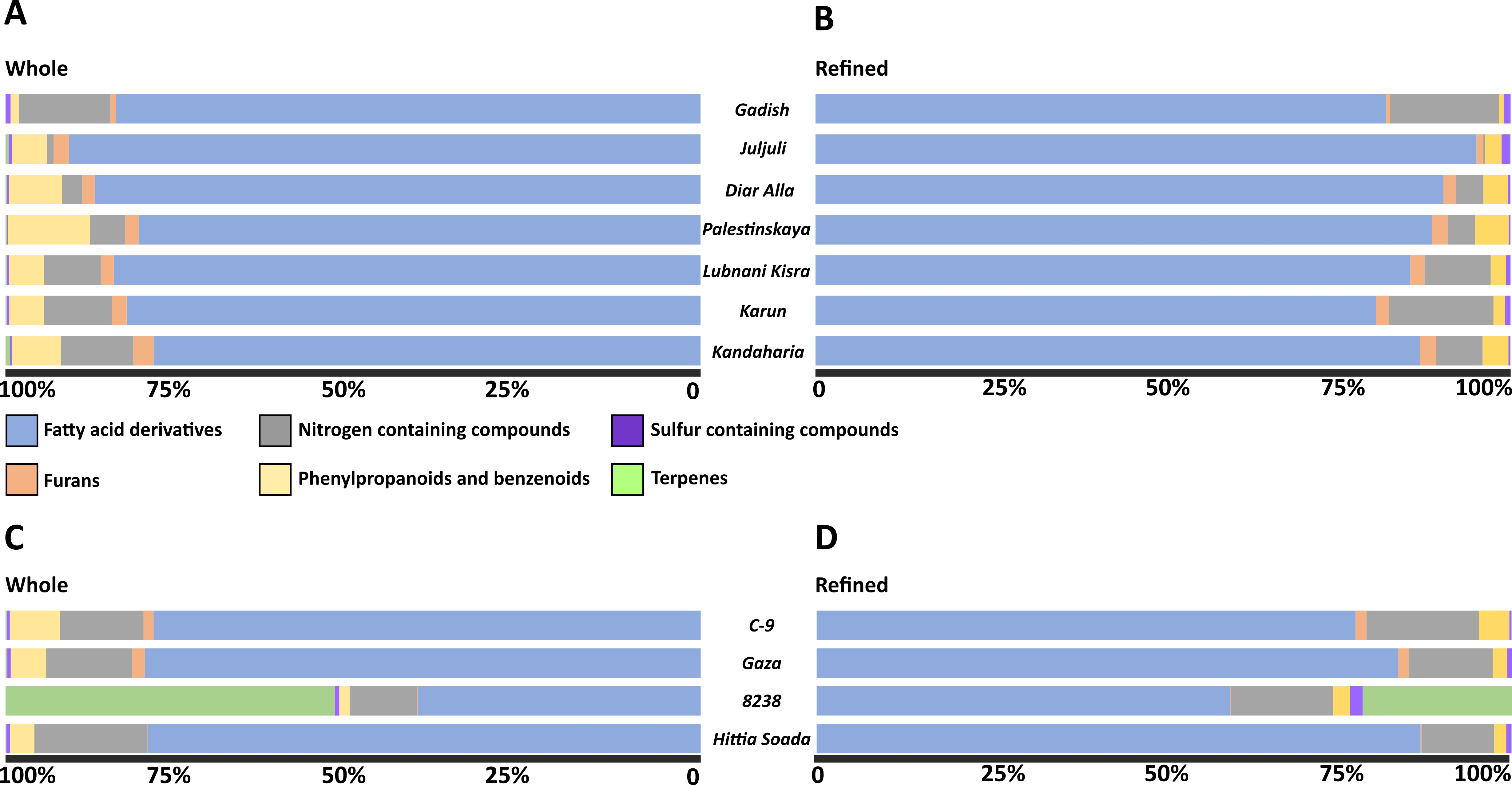

Supplement: Supplementary Figure S3 — Aroma class composition of bread wheat and durum flours (2019–2020). The compositions of aroma compounds in flours are presented as the relative proportion of each of the six main groups of volatiles. (A) Whole and (B) refined flours in lines of T. aestivum; (C) the corresponding flours of T. durum. [file Image_3.jpg]

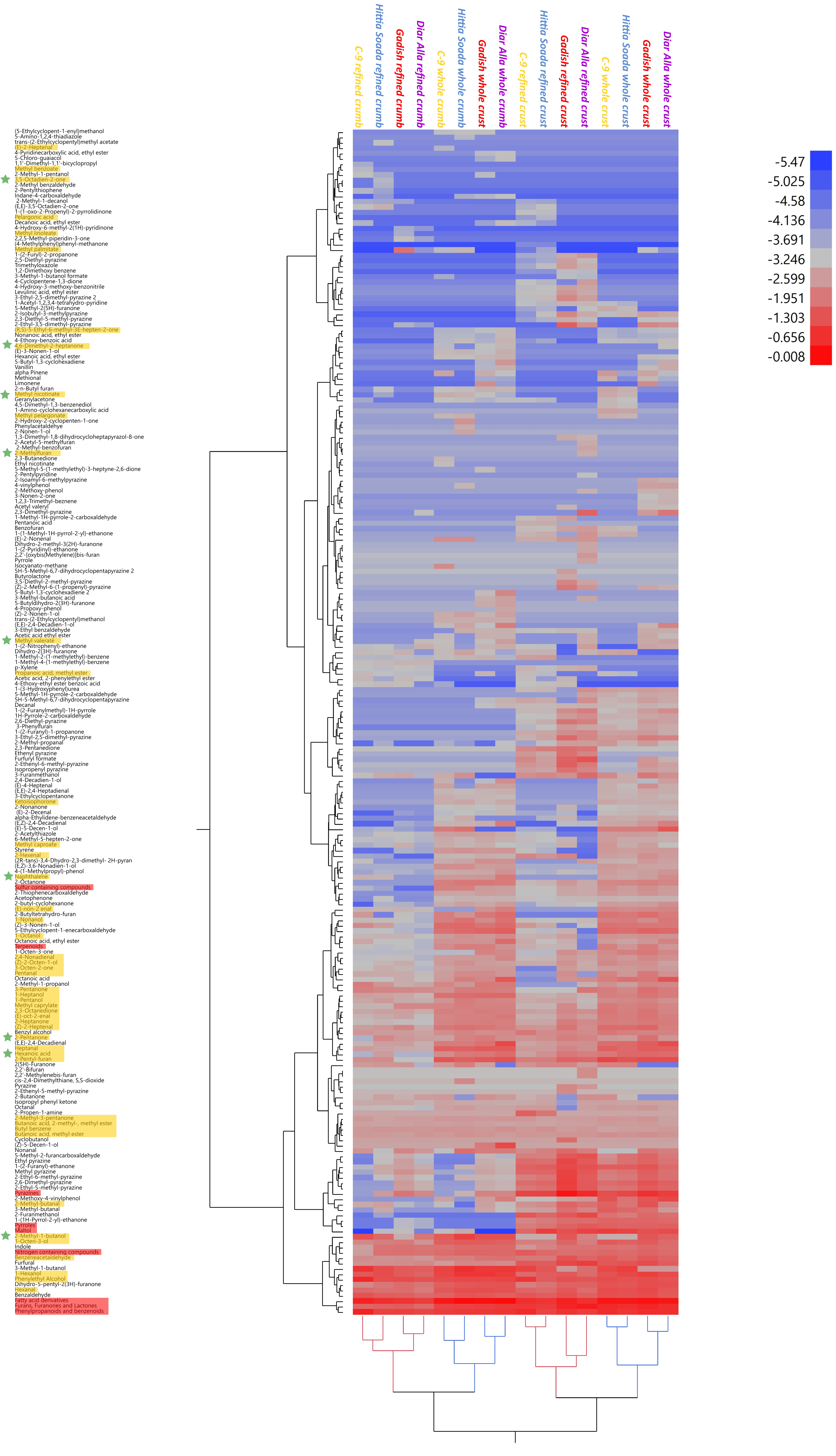

Supplement: Supplementary Figure S4 — Aroma and taste compounds in crumbs/crusts of breads prepared from landrace flours (2019–2020). Presented are 2-way hierarchical clustering models (Ward's method, data is log10 transformed, n = 3), using aroma compounds detected in crumbs and crusts of breads prepared from landrace lines (“Diar alla”/“Hittia soada”) or modern reference cultivars (“Gadish”/“C-9”). Metabolite concentrations are represented by an increasing color gradient from blue to red. Classes of aroma compounds are marked red; metabolites that were present in flours are marked yellow; those marked with green stars retained their increased levels in breads as well as in flours. Dendogram and hierarchical clustering analysis were generated and visualized using JMP (version 14.0.0). [file Image_4.jpg]

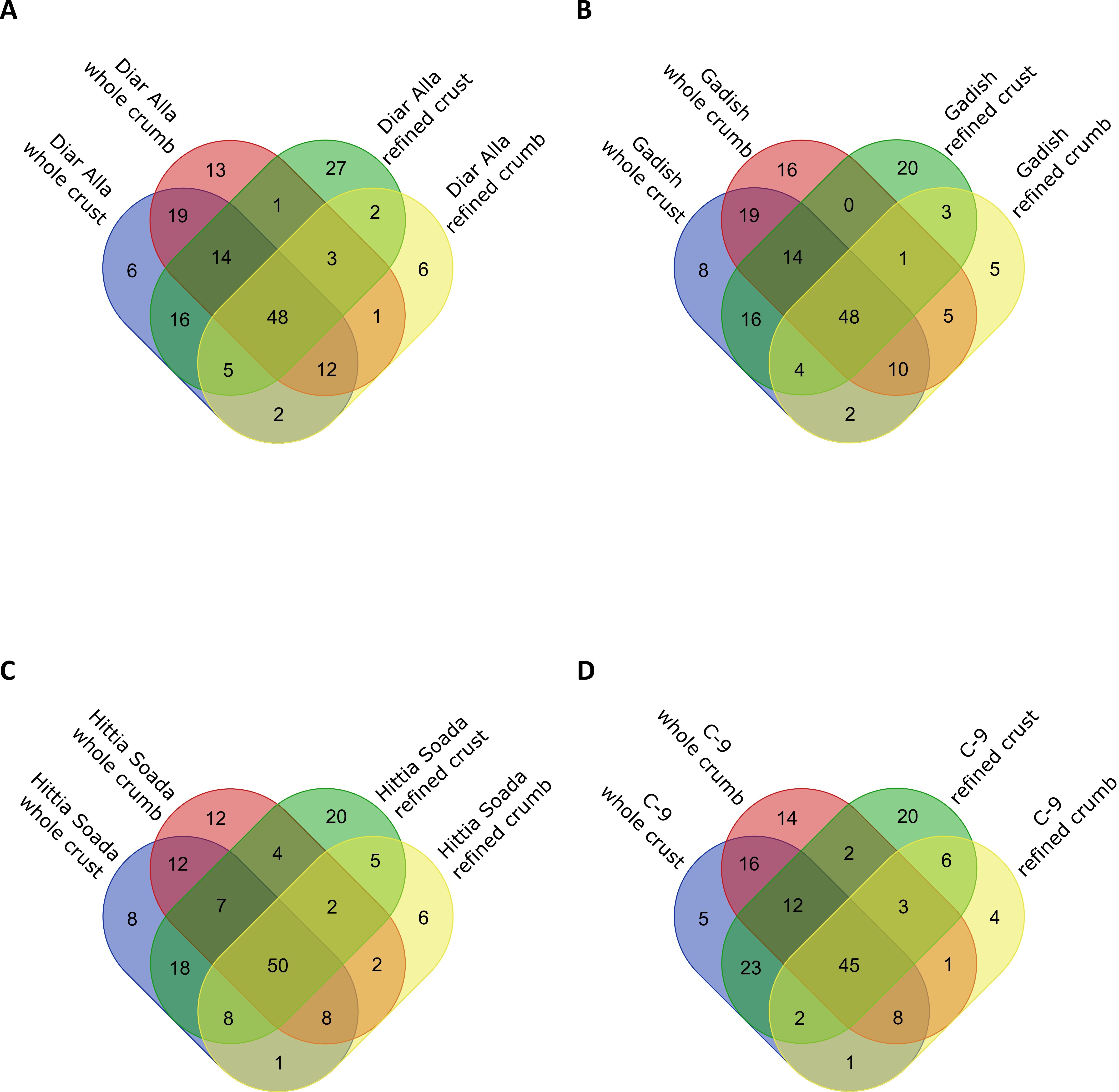

Supplement: Supplementary Figure S5 — Partitioning of volatile compounds across breads used in this work (2019–2020). Presented are Venn diagrams representing the partitioning of volatile compounds detected in crumbs and crusts from whole and refined bread wheat and durum lines: “Diar alla” (A), “Gadish” (B), “Hittia soada” (C) and “C-9” (D). Diagrams were prepared using the online University of Gent Venn diagram tool (www.bioinformatics.psb.ugent.be/webtools/Venn). [file Image_5.jpg]

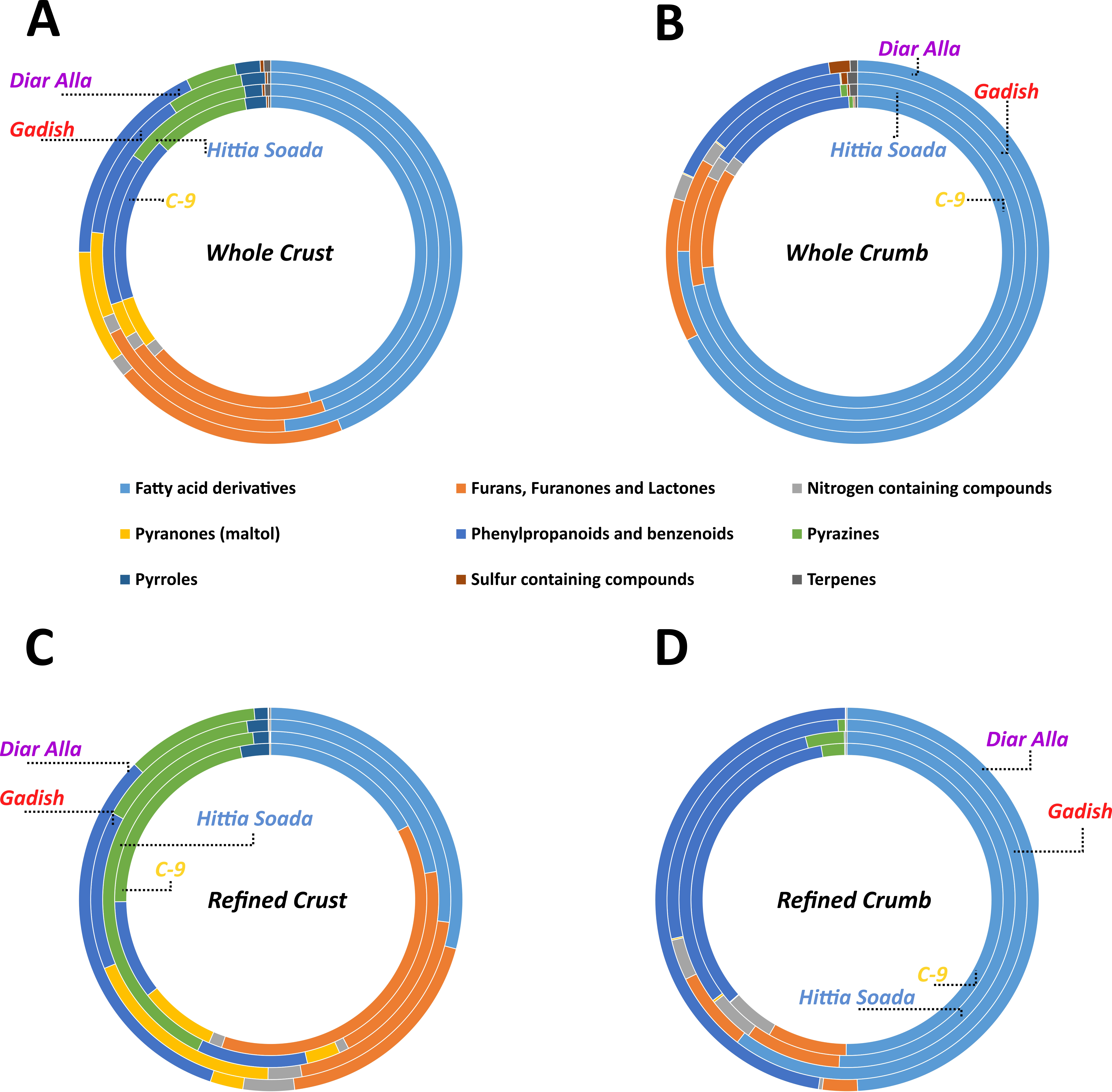

Supplement: Supplementary Figure S6 — Aroma class composition of bread wheat and durum breads (2019–2020). The compositions of aroma compounds are presented as the relative proportion of each of the nine main groups of volatiles detected in crumbs or crusts of breads prepared from whole and refined flours of modern and landrace lines, n = 3. Composition of (A) crusts and (B) crumbs of breads prepared from whole flours; (C, D) are the corresponding compositions of breads prepared from refined flours. [file Image_6.jpg]
